# The Role of Alexithymia in Social Learning and Feedback-Driven Social Inferences

**DOI:** 10.5334/cpsy.153

**Published:** 2026-03-19

**Authors:** Mina Hosseinnezhad, Soroosh Golbabaei, Mohammad Reza Bigham, Khatereh Borhani

**Affiliations:** 1 Institute for Cognitive and Brain Sciences, Shahid Beheshti University, Tehran, Iran; 2 Department of Psychiatry and Psychotherapy, Jena University Hospital, Jena, Germany; 3 German Center for Mental Health (DZPG), partner site Halle-Jena-Magdeburg, Jena, Germany

**Keywords:** alexithymia, social learning, emotion, reinforcement learning, drift-diffusion model

## Abstract

Dynamic social interactions and feedback are crucial for understanding others’ emotions, particularly when confronted with contradictory emotional cues. Alexithymia, a condition that co-occurs with many psychiatric disorders, is characterized by impairment in emotional processing. However, computational mechanisms by which it alters social inferences based on feedback cues remain unexplored. To examine this, 60 participants with low and high levels of alexithymia completed an emotional learning task involving contradictory social (verbal and visual) cues to infer targets’ emotions. Computational analyses, including bin-based, reinforcement learning, and drift-diffusion modeling, revealed how alexithymia alters latent parameters that govern value updating and choice. Individuals with high alexithymia demonstrated lower accuracy in learning from social feedback, and learning rate for verbal cues was negatively associated with difficulties in identifying and describing feelings. Drift diffusion analysis revealed a perceptual bias toward the visual cue, with higher drift rates and bias in the visual-correct condition, and a general requirement for greater evidence accumulation to infer others’ emotions. These findings suggest that individuals with high alexithymia exhibit impaired social learning and difficulty with decision-making in situations with conflicting social information, with computational modeling quantifying the latent processes involved and advancing mechanistic targets for computational psychiatry.

Emotional reactions, whether verbal or visual, during interpersonal communications, are crucial cues for interpreting others’ inner emotional state and feelings (Adolphs, 2002; Timler, 2003). However, the expression of emotions is not always clear, consistent, or easy to understand. This complexity, sometimes described as “smiling on the outside, crying on the inside” (Seibt et al., 2015), can make it challenging to accurately interpret emotional cues.

When a friend smiles and enthusiastically recounts birthday memories, we typically infer they are happy, and ask for more details. In contrast, if a friend’s expression remains neutral despite saying they enjoyed the party, their true feelings are more challenging to interpret. Relying solely on verbal cues, without taking non-verbal cues into account, can lead to misunderstandings. If they refuse to elaborate further, it highlights that facial expressions would have provided more accurate insight than verbal expressions alone. Through repeated interactions and feedback, people refine their understanding of emotional cues. As they improve, they learn to value informative cues and better predict others’ inner states (Zaki et al., 2016). This social reinforcement learning process enables individuals to acquire knowledge by observing others’ emotions, making inferences about those emotions, acting accordingly, and subsequently learning from the outcomes of their actions (Joiner et al., 2017; Sutton & Barto, 1994). Social reinforcement learning is vital for effective communication and fostering better relationships, whereas deficits in this process can impair interpersonal functioning (Vélez & Gweon, 2021; Zaki et al., 2016).

Despite its importance, social reinforcement learning has only recently been studied in a few cases. Recent evidence shows that social reinforcement learning enhances empathy towards out-group members (Hein et al., 2016) and increases moral outrage in online social networks (Brady et al., 2021). Conversely, deficits in social learning have also been reported in psychiatric disorders, such as depression (Frey et al., 2021; Frey & McCabe, 2020; Jin et al., 2023) and autism spectrum disorder (ASD; Zhao et al., 2024). These findings underscore the need for theoretical frameworks capable of explaining how atypical learning mechanisms contribute to social and emotional dysfunctions.

One such framework is predictive coding, which provides a powerful computational account of perception, learning, and decision-making. According to this account, the brain functions as a probabilistic inference system that strives to construct a stable model of the environment. When confronting uncertainty, it continuously compares prior beliefs or internal representations (priors) with incoming sensory inputs, and discrepancies between the two generate prediction errors or entropy. These errors prompt the brain to update its internal models, with the extent of updating determined by the precision weighting assigned to those errors (Friston, 2009; Gilbert et al., 2022).

Computational modeling approaches based on such theoretical frameworks, have proven invaluable for elucidating how disruptions in learning and decision-making contribute to the expression of symptoms, thereby providing insight into the mechanisms underlying these symptoms (Smith et al., 2021). Reinforcement learning models, for example, characterize how individuals update the value of choices based on feedback and have revealed that a psychiatric disorder such as depression is associated with a lower social learning rate and slower value updating (Frey et al., 2021; Frey & McCabe, 2020), or with reduced value updating more generally (Safra et al., 2019). On the other hand, Autism is explained by weak predictive priors, where every local information is treated as novel information leading to highly value updating (Happé & Frith, 2006; Mottron et al., 2006; Van De Cruys et al., 2014). Similarly, drift diffusion models by considering the dynamics of decisions have demonstrated diminished reward sensitivity and slower evidence accumulation in individuals with major depressive disorder (Shen et al., 2024). In ASD, Bayesian modeling has revealed that higher autistic traits are linked to reduced exploitation of social cues (Sevgi et al., 2020) and diminished flexibility in adjusting strategies during social interactions, leading to reduced social reciprocity (Forgeot d’Arc et al., 2020). Within the predictive coding framework, these findings have been interpreted as evidence for overly stable and negatively biased priors in depression, reduced or hypo-priors in ASD, and abnormal precision weighting in both disorders.

Although the application of computational models holds great promise for advancing our understanding of social learning difficulties in psychiatric disorders, many important issues remain to be addressed. In particular, alexithymia, a condition characterized by difficulties in discriminating, identifying and describing one’s own and others’ emotions (Nemiah et al., 1976), shows a high degree of comorbidity with both depression (Hintikka et al., 2001; Honkalampi et al., 2000) and ASD (Bird & Cook, 2013; Hill et al., 2004). Yet, to date, no study has specifically examined social learning in relation to alexithymia.

Alexithymia is linked to difficulties in processing verbal and visual emotional stimuli (Grynberg et al., 2012; Herbert et al., 2011; Lane et al., 1996; Montebarocci et al., 2011; Parker et al., 1993; Parker et al., 2005). It also impairs learning the emotional value of conditioned stimuli during Pavlovian threat conditioning (Starita et al., 2016). Individuals with higher levels of alexithymia need more time to learn the value of aversively motivated actions in instrumental learning (Starita & Di Pellegrino, 2018). At the electrophysiological level, they display a limited amplitude of feedback-related negativity (FRN) in response to conditioned stimuli with unexpected feedback during Pavlovian appetitive conditioning, though FRN responses to expected feedback remain unaffected (Starita et al., 2019). Cumulatively, these findings reveal that alexithymia hampers constructing mental representations of emotional stimuli and utilizing them for non-social learning (Starita et al., 2018; Starita et al., 2019). Yet, it is unknown whether individuals with high alexithymia (HA) have similar challenges when learning the value of social stimuli and cues.

Social cues play an important role in interpersonal communication, enabling individuals to predict others’ internal and emotional states and utilize this understanding to enhance social cognitive skills. Social cues are conveyed through verbal behaviors, such as spoken language or text, as well as through non-verbal behaviors like facial expressions, gestures, and prosody, all of which provide information for identifying others’ emotional states (Polzin, 2000; Welding & Samur, 2018). Previous findings are inconsistent regarding whether alexithymia is associated with difficulties in identifying emotions across verbal and non-verbal modalities. Some studies have reported no impairments in judging emotions or prosody from auditory stimuli (Swart et al., 2009; Telli & Bilge, 2024), whereas others have documented deficits in perceiving emotions from such stimuli (Goerlich et al., 2012; Goerlich-Dobre et al., 2014). In contrast, evidence from non-verbal bodily and facial expressions is more consistent, indicating that higher levels of alexithymia are mostly associated with reduced accuracy in recognizing emotions from visual non-verbal cues (Borhani et al., 2016; Grynberg et al., 2012; Jongen et al., 2014). Importantly, it remains unclear how potential difficulties in inferring emotions from social signals may impact social learning in individuals with alexithymia.

Hence, the current study was designed to investigate whether individuals with alexithymia show impairment in learning from the value of emotional cues and feedback in the emotional learning task. For this purpose, individuals with high and low levels of alexithymia participated in the emotional learning task (Zaki et al., 2016), and two computational modeling approaches, namely reinforcement learning (RL) and drift-diffusion, were employed to investigate social learning in alexithymia. We hypothesize that individuals with high alexithymia (HA) compared to low alexithymia (LA) individuals will exhibit reduced learning, evidenced by fewer correct responses and lower estimated learning rates. We further predict that, in conflicting social contexts, they will require greater information accumulation before selecting a correct social inference. Although the comparison across cue modalities is exploratory and prior evidence is limited, we expect the high-alexithymia group to rely more on salient visual cues than on verbal cues.

## Methods

### Participants

Participants were university students recruited through various channels, including face-to-face interactions, advertisements on social networking platforms (i.e., LinkedIn, Instagram, Telegram, and Twitter), and notices distributed in universities and dormitories. Those interested were directed to an online survey platform (Porsline), where they filled out the Farsi version of the Toronto Alexithymia Scale (TAS-20; Besharat, 2007). In total, 1,080 volunteers completed the questionnaire, among whom 78 obtained scores falling within the low and high alexithymia ranges and were subsequently invited to the laboratory to participate in the experiment. Inclusion criteria were the absence of self-reported neurological abnormalities or psychological disorders. Considering the established connection between Alexithymia and Depression (Allen et al., 2011; Hintikka et al., 2001; Honkalampi et al., 2000), participants also completed the Farsi version of the Beck Depression Inventory-II (Ghassemzadeh et al., 2005), and those with a Beck score of above 20 were excluded from further participation (Vanheule, Desmet, Verhaeghe, et al., 2007). Moreover, as in Zaki et al. (2016), data from participants who relied on only one social cue to assess the emotional states of the target (n = 8; see emotional learning task section), or not adhere to instructions (n = 10) were excluded, resulting in a total of 18 exclusions. The final sample consisted of 30 participants in the low alexithymia (LA) group (female: 16, *M*_age_: 25.37 years, *SD*_age_ = 4.35, range = 18–35 years) with TAS-20 score of ≤ 36, and 30 participants in the high alexithymia (HA) group (female: 16, *M*_age_: 23.12 years, *SD*_age_ = 3.09, range = 18–35 years) with TAS-20 score of ≥ 61 (Franz et al., 2004). The sample size was determined using a G*Power 3.1 power analysis (Faul et al., 2007). For a mixed-model ANOVA with an alpha level of 0.05, an effect size of 0.3, and a power of 0.85, at least 58 participants were required. To account for potential participant exclusions due to task misunderstanding, the final sample consisted of 60 participants. Written informed consent was obtained from participants, and the study was approved by the ethics committee of the Shahid Beheshti University.

### Measures

#### Toronto Alexithymia Scale (TAS-20)

TAS-20 (Bagby et al., 1994) is a 20-item self-report measure of alexithymia, composed of three subscales of difficulty identifying feelings (DIF), difficulty describing feelings (DDF), and externally oriented thinking (EOT). Each item is responded to on a 5-point Likert scale (1 = strongly disagree; 5 = strongly agree), yielding a total score within the range of 20 to 100. A score of 36 or below indicates LA, while scores of 61 or above are considered as HA (Borhani et al., 2016; Franz et al., 2004). In this study, we utilized the validated Farsi version of the TAS-20 (Besharat, 2007), which has demonstrated high internal consistency with Cronbach’s Alpha of .85, .84, .80, and .87 for DIF, DDF, EOT, and total-items, respectively.

#### Beck Depression Inventory-II (BDI-II)

BDI-II (Beck et al., 1996) is a 21-item self-report measure that evaluates the severity of depression experienced over the preceding two weeks. Each item is rated on a four-point scale (0 = absent or mild symptom; 3 = severe symptom). The total score ranges from 0 to 63, where higher scores indicate greater severity of depression. In this study, the BDI-II cut-off points were defined as follows: scores of 1–10 indicating normal mood, 11–16 suggesting mild mood disturbance, 17–20 indicating borderline clinical depression, 21–30 suggesting moderate depression, and scores over 40 indicating extreme depression. The Farsi version of the BDI-II, validated by Ghassemzadeh et al. (2005), was used in this study. This version has demonstrated a high internal consistency with Cronbach’s Alpha of .87.

#### Interpersonal Reactivity Index (IRI)

The 16-item Farsi version of IRI (Golbabaei, Barati, et al., 2023) was used in this study, a self-report measure of empathy, composed of four subscales of Perspective taking (PT), Fantasy (F), Personal distress (PD), and Empathic concern (EC). Each item is responded to on a 5-point Likert scale (0 = does not describe me well, 4 = it describes me well). This validated short Farsi version of IRI is reported to have a good internal consistency with Cronbach’s Alpha in the range of .67–.71.

### Stimuli

The stimuli utilized in this study consisted of both visual (video clips) and verbal (sentences) components, created based on the methods used in the previous study (Zaki et al., 2008).

#### Visual Stimuli

To create video clips eliciting happy and sad emotions, eight individuals (four females; *M_age_* = 26.12; *SD_age_* = 1.53) were asked to recall and write down one of the most negative (e.g., loss of a parent) and positive (e.g., marriage) events in their lives. After providing written consent for the use of their videos, they sat in front of a camera to talk about the chosen events. The videos were framed to capture the participant’s face and upper shoulders. A total of 16 videos were recorded, equally split between positive and negative events, with durations ranging from two minutes and 15 seconds to three minutes. To select the final set of video targets, 20 additional individuals (12 female; *M_age_* = 25.54, *SD_age_* = 2.20) rated these videos on a 9-point Likert scale (1 = very low, 9 = very high), assessing the intensity of six basic emotions (happiness, sadness, anger, fear, surprise, and disgust). They also used the Self-Assessment Manikin method (Bradley & Lang, 1994) to rate the valence (–4 = extremely unpleasant, +4 = extremely pleasant) and arousal (1 = calm and 9 = excited) dimensions of the videos. Based on these ratings, three positive and three negative silent video clips featuring three social targets (two females) were selected. The selection criteria were the mean ratings of higher than seven for the corresponding emotion (happiness/sadness) and lower than five for other emotions. Each video clip was then divided into 24 five-second segments, resulting in a total of 144 videos evenly distributed between three targets and two emotions. To ensure the reliability of these ratings, an inter-rater reliability analysis was conducted, which showed excellent consistency across raters for all stimuli (all average Intraclass Correlation Coefficients (ICCs) > .96; see the Supplementary Material File 1; Table S1).

#### Verbal Stimuli

We presented verbal stimuli as written text similar to Zaki et al. (2016). By using text, we ensured that every participant received the same information, avoiding differences in tone or intonation that might unintentionally affect emotional recognition. This way, we could focus specifically on how people learn from the emotional content itself.

A set of 172 Farsi sentences describing emotional events (half positive, half negative) was prepared by the first author. These sentences were rated by a separate group of 20 participants using the same rating scale procedure. Sentences were selected based on mean ratings greater than five for the intensity of positive or negative emotion. Based on these ratings, 72 positive sentences (*M* ≥ 5.8) and 72 negative sentences (*M* ≥ 5.4) were chosen. The positive (*M* = 3.8; *SD* = .98) and negative sentences (*M* = 3.81; *SD* = 1.05) did not differ with respect to word count, *t* = .40, *p* = .68.

### Emotional Learning Task

**Figure 1:**
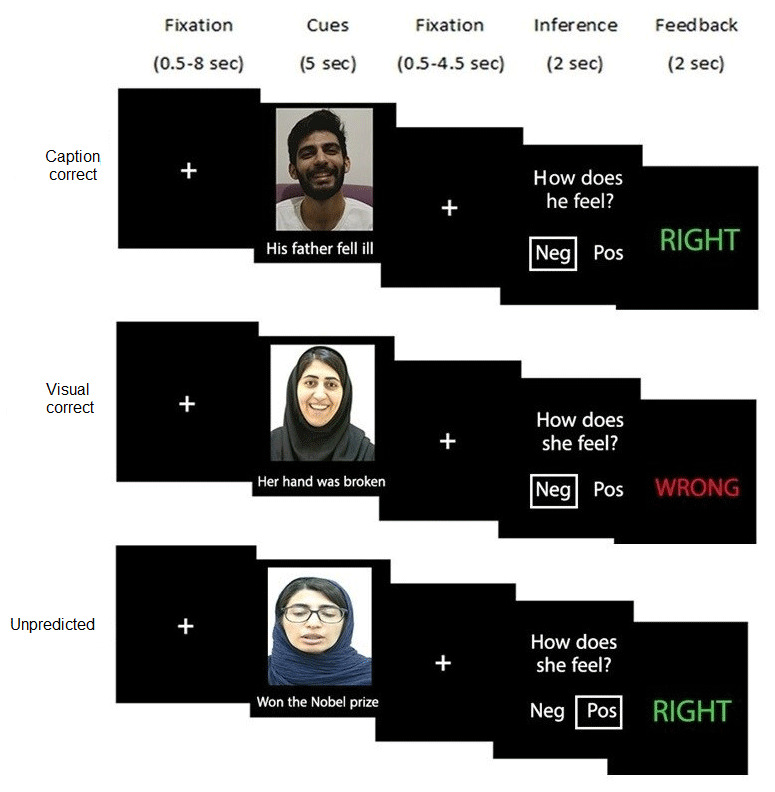
Schematic representation of emotional learning task. *Note:* Each trial began with a fixation cross (jittered time between 0.5–8 sec), followed by incongruent verbal and visual cues from three social targets. After a second fixation cross (jittered time between 0.5–4.5 sec), the question was presented to the participants. Participants were instructed to use the cues to guess the feelings of the targets and then receive feedback on whether their responses were correct or not. Each target (caption-correct and visual-correct) was coded to a specific cue, verbal and visual, respectively, that produced correct feedback, but for the third target (unpredicted), none of the cues could produce the correct feedback.

The Emotional Learning Task, originally introduced by Zaki et al. (2016), investigates how participants use feedback to infer the target’s internal states and emotions when presented with conflicting verbal and visual cues. The task consists of 144 trials, with three social targets (48 trials each) concurrently displaying incongruent verbal and visual cues. Specifically, 24 positive videos from each target are paired with 24 negative captions, and vice versa, randomly. For example, a video in which the target appears to feel negative is paired with a positive caption, or the reverse. Each stimulus is presented only once to the participants. Each social target is matched with a pseudorandomized combination of visual-correct, caption-correct, or unpredicted feedback. Each trial begins with a jittered fixation of 500–8500 ms, followed by a 5000 ms stimulus presentation. After another jittered fixation of 500–4500 ms, the question “How does the target feel?” appears for 2000 ms, prompting participants to infer whether the target’s emotion is positive or negative. Participants respond by pressing the right and left keys for positive and negative responses, with the order of responses counterbalanced between participants. Feedback is provided for 2000 ms, indicating whether their choice was correct or incorrect (Figure 1).

Feedback is structured differently for the three social targets, visual-correct, caption-correct, and unpredicted targets, randomly ascribed to each social target. For the visual-correct target, the visual cue is correct on 87.5% of trials (42/48), while the verbal cue is correct on the remaining 12.5% of trials (6/48). Conversely, for the caption-correct target, the verbal cue is correct on 87.5% of trials (42/48), with the visual cue being correct on 12.5% (6/48). In the unpredicted condition, neither verbal nor visual cue reliably predicts the target’s emotional states.

### Procedure

The Farsi version of the 20-item Toronto Alexithymia Scale (TAS-20; Besharat, 2007) and the Beck Depression Inventory (BDI-II; Ghassemzadeh et al., 2005) were administered to assess participants’ levels of the Alexithymia trait and depressive symptoms, respectively. Individuals who met the inclusion criteria were invited to the laboratory. Upon arrival, participants were seated in a comfortable chair in front of a laptop screen where they signed the informed consent form. After the provision of both oral and written instructions, participants completed the Emotional Learning Task. Subsequently, they filled out the IRI (Golbabaei, Barati, et al., 2023).

### Bin-Based Analysis

In order to assess participants’ learning over time, 48 trials of each condition (visual-correct, caption-correct, and unpredicted targets) were divided into four “bins” of 12 trials each. The number of correct responses was calculated for each bin in each condition. A 2 × 3 × 4 mixed ANOVA was conducted, with group HA and LA as the between-subject factor, and condition (visual-correct, caption-correct, and unpredicted), and block (Bins 1 to 4) as within-subject factors, to compare emotional learning accuracy across conditions between two groups. Post-hoc t-tests with Bonferroni correction were used to account for multiple comparisons. Moreover, Pearson correlations were conducted to examine the relationships between the number of correct responses in each condition (as an indicator of emotional learning) and the TAS-20 score, its subscales, as well as the IRI-16 score and its subscales.

### Reinforcement Learning model

We utilized six Q-Learning models to compute the emotional learning rate of each participant and to compare groups with regard to emotional learning rate. In general, participants first start with an initial expected value of .5 for each cue, which means that they value visual and verbal cues the same. Then, based on the option selected on each trial and the feedback received (*r_t_*), prediction error (*δ_t_*) was computed as the discrepancy between the obtained reward and the expected value of the chosen response (*Q_i, t–1_*). The expected value of the chosen response was then updated according to Equation 1.


1 \[
{Q}_{i, t} = {Q}_{i, t-1} + {\alpha}_{i} \times ({r}_{t} - {Q}_{i, t-1})
\]

where *Q_i,t_* is the value of the chosen action (*i*) in trial *t, α* is the learning rate by which the values for the actions are updated, and *r_t_* is the feedback that is shown to the participant in trial *t*. Then, the probability of choosing an option is computed according to the softmax distribution (Daw & Doya, 2006):


2 \[
{{P}_{i, t}} \approx exp\left(\beta \left({{Q}_{i, t}}\right)\right)/{\sum}_{i = 1}^{k}exp \left(\beta \left({{Q}_{i, t}}\right)\right)
\]

where β is an inverse softmax temperature parameter representing choice stochasticity, such that lower values reflect more random and exploratory choices, whereas higher values indicate more deterministic, value-driven behavior, leading individuals to preferentially choose the option with the higher expected value (Sutton & Barto, 1994; Zaki et al., 2016).

The six models differed in two respects: (1) whether the learning rate (α) was shared across conditions or allowed to vary by condition, (2) whether learning was symmetric or split by the sign of the prediction error, (3) whether initial expected values (priors) were fixed at .5 or freely estimated as a parameter. For the first set of reinforcement learning (RL) models (models 1–3), we used a single learning rate to update the values associated with both the caption and the visual target (condition) for each participant. To further examine the potential differences in learning dynamics across cues, we implemented an alternative two-learning-rate model, in which separate learning rates were estimated for the visual and caption targets (models 4–6). In addition, for each of these sets, we also fit a variant with separate learning rates for positive and negative prediction errors (α^+^ and α^–^; prediction errors defined as r_t_ – Q_i, t–1_). Finally, we fit an additional variant in which the initial values Q_i,0_ were freely estimated, allowing them to capture participants’ priors (as in prior work; Kozakevich Arbel et al., 2024). Thus, model-1 includes a single learning rate (α), and initial values for options are fixed to .5. Model-2 includes a single alpha for both conditions, but separate learning rates for positive and negative prediction errors (α^+^, α^–^). Model-3 includes a single alpha for both conditions, but the initial values of choices are freely estimated. Model-4 includes a separate learning rate per condition (α^visual^ and α^caption^) and initial values for options are fixed to .5. Model-5 contains four different learning rates, corresponding to different conditions and prediction errors (α^–visual^, α^+visual^, α^–caption^, α^+caption^), and initial values for options are fixed to .5. Model-6 contains a separate learning rate per condition (α^visual^ and α^caption^) and initial values for each option are also freely estimated. See the Supplementary Material File 4 and also previous studies (Daw et al., 2002; Frank et al., 2007; Gershman, 2015) for a full and detailed description of these model variants.

Python’s SLSQP was used for optimization, with 50 different starting points. Additionally, the learning rate (α) and prior were bounded between 0 and 1, and the inverse temperature (β) was bounded between 0 and 20. Using the mentioned procedure, we assessed the learning rate of each cue as well as stochasticity of decisions for each participant.

In order to choose the best model, we calculated the Bayesian information criterion (BIC; Neath & Cavanaugh, 2012) to compare the models. The results revealed that BIC favored the model with two learning rates without distinguishing between positive and negative prediction errors, and fixed initial values of .5 (model 4; see Supplementary Material File 4 for the details). We also conducted a parameter recovery analysis to assess the reliability of parameter estimations (as suggested in Wilson & Collins, 2019). Simulated datasets were generated over true learning rates (α*_Visual_* and α*_Caption_*) and inverse temperature (β) values. Each dataset was fit using the same procedure applied to participants’ data. Recovery was quantified using the Pearson correlation between true and recovered parameters, and we found a strong correlation between them (All *r’*s > .70; see Supplementary Material File 4 for a detailed description of parameter recovery).

### Drift Diffusion Model of Decision-Making

To model social learning and decision-making processes by accounting for both response accuracy and reaction time, a diffusion model was employed. The diffusion model was fit using fast-dm (Voss et al., 2015; Voss & Voss, 2007) with Kolmogorov-Smirnov parameter optimization. Drift rate, boundary separation, and relative starting point were allowed to vary freely across the three conditions, while all other parameters were kept fixed across conditions. In each condition, the upper decision boundary was assigned to correct responses, while the lower decision boundary corresponded to incorrect responses. Notably, in the visual-correct condition, the correct response (upper boundary) was defined as responding based on the visual cue, whereas in the caption-correct condition, the correct response was based on the verbal cue. The relative starting point indicates bias toward one of the options, with a higher starting point reflecting a stronger bias toward the correct response in each condition. Boundary separation represents the amount of information required to make a decision, with higher values indicating more conservative decision-making. Lastly, the drift rate reflects the speed of information accumulation toward a decision, with positive values corresponding to correct responses and negative values indicating incorrect responses. While the primary aim is to examine these three measures across groups and conditions, based on previous literature linking alexithymia to slower response latencies (De Galan et al., 2014; Fuchs et al., 2024; Ihme et al., 2014; Reker et al., 2010; Starita & Di Pellegrino, 2018; Suslow et al., 2024; Zhang et al., 2011), we will also explore group differences in both duration of non-decisional processes and the speed of response execution. These analyses will not consider differences across conditions, as no prior evidence suggests such effects, and the conditions provide the same amount and type of information (i.e., both visual and caption information).

## Results

### Bin-Based Analysis

**Figure 2:**
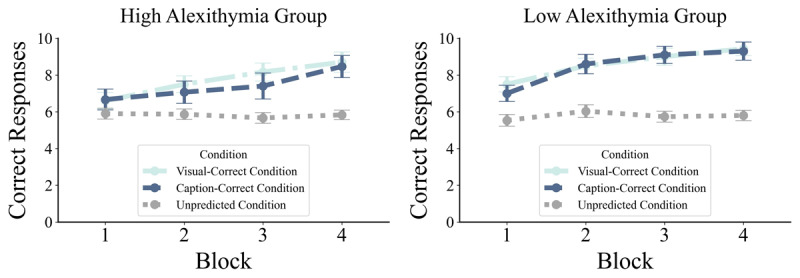
Interaction Between Groups, Conditions, and Blocks in Average of Correct Responses. *Notes:* Improving social learning accuracy from caption-correct and visual-correct conditions across blocks in high Alexithymia and low alexithymia groups, while, learning accuracy in unpredicted-condition did not improve. Error bars represent standard error of the mean.

First, a 2 (Group: low alexithymia [LA] vs. high alexithymia [HA]) × 3 (Condition: visual-correct, caption-correct, unpredicted) × 4 (Block) repeated-measures mixed ANOVA was conducted on the number of correct responses (Table 1). The results revealed a significant Condition × Block interaction, *F*(5.05, 292.98) = 4.731, *p* < .001, *η2* = .075, while no other interactions reached significance (*p* > .05; Table 1). Using paired t-test as the post-hoc test, we found significant differences in all comparisons of learning accuracy across conditions and blocks (all .001 < *p’s* ≤ .042), except for comparisons between the visual-correct condition in third and fourth blocks, *t*(59) = –1.605, *p* = .114, *Cohen’s d* = –.207, and between caption-correct condition in second and third blocks, *t*(59) = –1.284, *p* = .204, *Cohen’s d* = –.166 (Supplementary Material File 1: Table S2). The results also revealed a significant main effect of group, *F*(1, 58) = 4.756, *p* = .033, *η2* = .076, indicating a higher rate of correct responses in the LA Group (*M* = 7.628, *SD* = 1.139) compared to HA Group (*M* = 6.989, *SD* = 1.139; Figure 2). A significant main effect of block, *F*(3, 174) = 16.656, *p* < .001, *η2* = .223, was also found, indicating changes in the number of correct responses over time. Using paired t-test as the post-hoc test, we found significant differences between all blocks (all .001 < *p’s* ≤ .007), except for comparisons between the second and third blocks, *t*(59) = –1.512, *p* = .136, *Cohen’s d* = –.195, and between the third and fourth blocks, *t*(59) = –2.234, *p* = .177, *Cohen’s d* = –.288 (Supplementary Material File 1: Table S3). Moreover, there was a significant main effect of Condition, *F*(1.67, 96.81) = 29.400, *p* < .001, *η2* = .336. Pairwise comparison showed significant differences between both the visual-correct, *t*(59) = 8.825, *p* < .001, *Cohen’s d* = 1.139, and caption-correct, *t*(59) = 6.105, *p* < .001, *Cohen’s d* = .788, conditions with unpredicted conditions, and no significant differences between visual-correct and caption-correct conditions, *t*(59) = .566, *p* = .574, *Cohen’s d* = .073 (Table 2). Moreover, we conducted a set of mixed-effects models using trial-by-trial accuracy as the learning measure. The results closely mirrored those obtained with the ANOVA (Supplementary Material File 2: Tables S1–S2).

**Table 1:** Mixed Anova Results for the Bin-Based Analysis of Social Learning.


VARIABLE	*df*	*F*	*P*	*η^2^_partial_*

Group	1, 58	4.756	.033	.076

Condition	1.67, 96.81	29.400	< .001	.336

Condition*Group	1.67, 96.81	1.550	.220	.026

Block	3, 174	16.656	< .001	.223

Block*Group	3, 174	1.056	.369	.018

Condition*Block	5.05, 292.98	4.731	< .001	.075

Condition*Block*Group	5.05, 292.98	.510	.800	.009


**Table 2:** Means, Standard Deviations, and t-test Results for Pairwise Comparisons of Learning Accuracy in Conditions.


CONDITION	*M*	*SD*	*t*	*p*	*Cohen’s d*

Visual-correct – Caption-correct	.900	12.323	.566	.574	.073

Visual-correct – Unpredicted	9.517	8.353	8.825	< .001	1.139

Caption-correct – Unpredicted	8.617	10.933	6.105	< .001	.788


**Figure 3:**
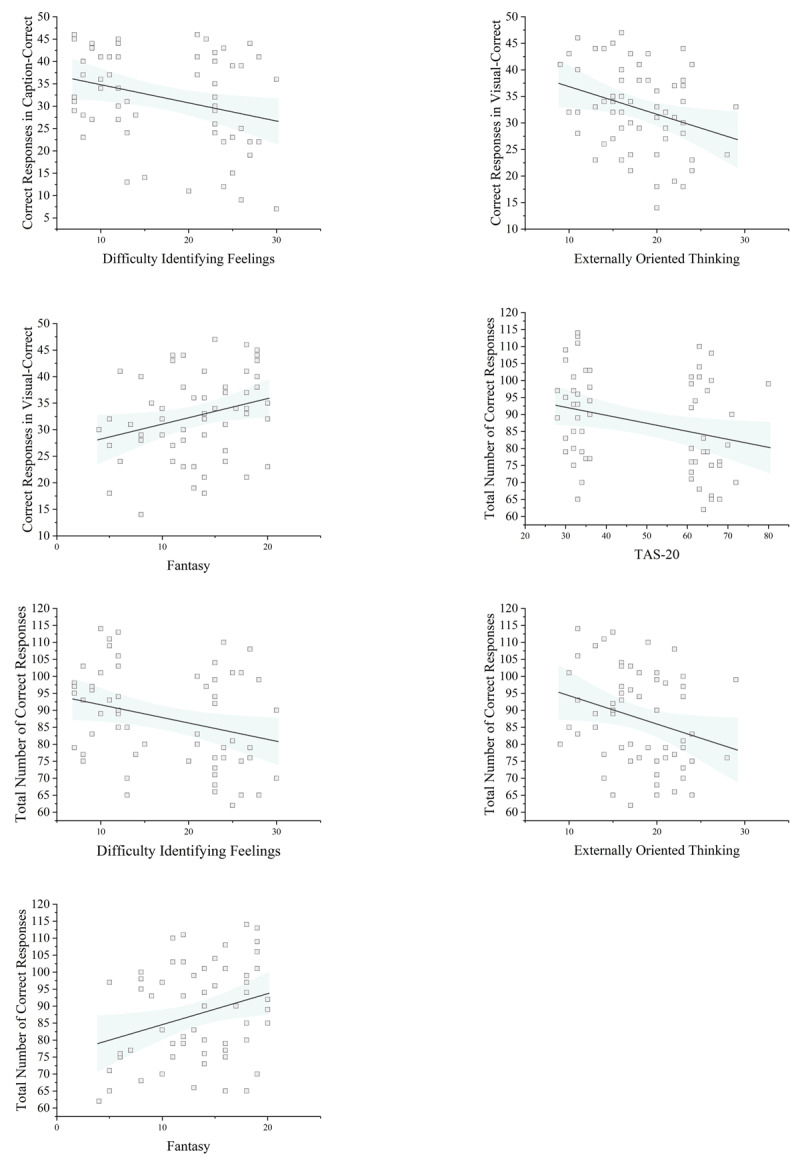
Correlation of TAS-20 and IRI Subscale Scores with Condition-Specific and Overall Response Accuracy. *Notes*: The shaded region represents the 95% confidence interval for the regression line. The discontinuity in TAS-20 scores reflects the purposive sampling of participants with high and low alexithymia (see Participants section for details).

To assess the association between learning accuracy and questionnaires (TAS-20, IRI, and their subscales), we evaluated the correlation between questionnaire scores and the number of correct responses in each condition and overall (Supplementary Material File 1: Table S4). A negative relationship was found between the number of correct responses in the visual-correct condition and EOT, *r* = –.292, *p* = .023, as well as the number of correct responses in the caption-correct condition and DIF, *r* = –.294, *p* = .023. Additionally, total correct responses were negatively correlated with TAS-20 score, *r* = –.297, *p* = .031, the DIF, *r* = –.287, *p* = .029, EOT, *r* = –.269, *p* = .038, and fantasy, *r* = –.292, *p* = .024. Moreover, a positive relationship was found between the number of correct responses in visual-correct condition and fantasy, *r* = .275, *p* = .034 (See Figure 3).

In addition, to account for the shared variance between alexithymia and IRI, as well as among their subscales, we estimated several mixed-effects models. First, we examined the effects of TAS-20 and IRI within the same model. Next, we included TAS-20 alongside all IRI subscales in a separate model, and finally, we tested the effects of TAS-20 subscales in another model. Overall, the results closely resembled those obtained from the correlational analyses (Supplementary Material File 3: Tables S1–16). Predicting accuracy, TAS-20 × Condition interactions were significant for both visual-correct, *t*(619) = 3.119, *p* = .002, and caption-correct, *t*(619) = –2.256, *p* = .024, conditions, indicating that TAS-20 scores differentially related to performance across these conditions. By contrast, in line with the correlational analysis, IRI × Condition interactions were not significant (all *p’s* > .05; Supplementary Material File 3: Tables S3–4). When including TAS-20 alongside all IRI subscales, two differences were also observed between the results of the mixed-effects models and the correlational analyses. In the subscale models, we observed a significant Personal Distress × caption-correct interaction, *t*(619) = –2.345, *p* = .019. However, the correlation between Personal Distress and correct responses in the caption-correct condition did not reach significance, although it showed a similar negative trend, *r*(58) = –.241, *p* = .063. In contrast, although Fantasy was significantly correlated with the total number of correct responses, *r*(58) = –.292, *p* = .024, neither the Fantasy × visual-correct interaction, *t*(619) = 1.708, *p* = .088, nor the Fantasy × caption-correct interaction, *t*(619) = 0.900, *p* = .368, was significant (Supplementary Material File 3: Table S5). Moreover, when TAS-20 subscales were used to predict accuracy, we found a significant interaction of caption-correct condition and DIF, *t*(640) = –3.981, *p* < .001, and DDF, *t*(640) = 2.440, *p* = .014, and significant interaction of visual-correct condition and EOT, *t*(640) = –2.501, *p* = .012 (Supplementary Material File 3: Tables S15–16).

### Reinforcement Learning

**Figure 4:**
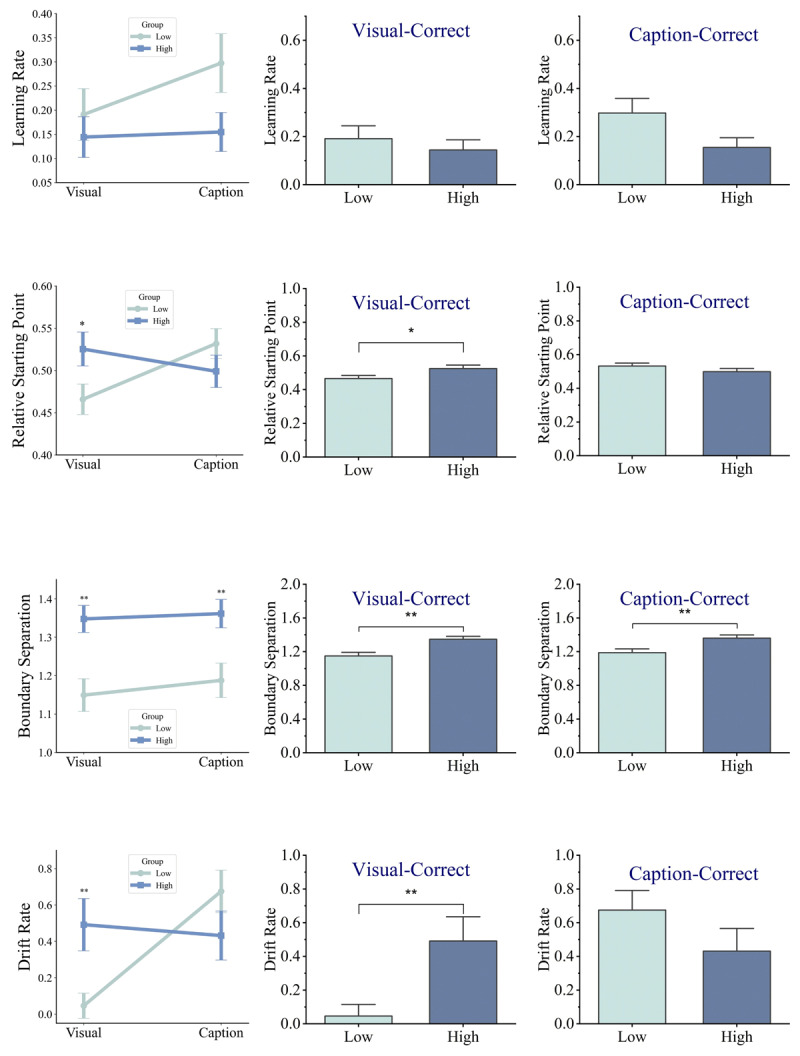
Comparison of Learning Rates and Drift-Diffusion Parameters Across Groups and Conditions. *Note*: Error bars represent standard error of the mean; *: *p* < .05; **: *p* < .001.

2 × 2 repeated measure ANOVA was conducted on learning rates extracted from the reinforcement learning with group (HA, LA) as the between-subjects factor and condition (visual-correct, caption-correct) as the within-subjects factor. The analysis of learning rates in the Q-Learning model revealed no significant interaction between group and condition, *F*(1,58) = .949, *p* = .334, *η2* = .006. Additionally, there was no significant main effect of group, *F*(1, 58) = 3.510, *p* = .066, *η2* = .057, nor condition, *F*(1, 58) = 1.411, *p* = .240, *η2* = .024 (Figure 4). Because we had no a priori hypothesis regarding group differences in stochasticity (the β parameter in the reinforcement learning model), we examined this parameter in an exploratory analysis using an independent-samples t-test. The high Alexithymia group (*M* = 7.483, *SD* = 8.354) showed slightly lower stochasticity than the low Alexithymia group (*M* = 8.013, *SD* = 8.229). However, this difference was not statistically significant, *t*(58) = 0.248, *p* = .805.

### Drift Diffusion

An ANOVA on starting points showed a significant interaction between group and condition, *F*(1,58) = 7.559, *p* = .008, *η2* = .115, but no significant main effect of group, *F*(1, 58) = .422, *p* = .518, *η2* = .007, or condition, *F*(1,58) = 1.390, *p* = .243, *η2* = .023. Post-hoc comparison using an independent t-test indicated a significant difference in starting point values between HA and LA groups in the visual correct condition, *t*(58) = 2.201, *p* = .032, *Cohen’s d* = 0.568, but not in the caption correct condition, *t*(58) = –1.264, *p* = .211, *Cohen’s d* = –.326. For the boundary separation parameter, however, no significant interaction, *F*(1,58) = .214, *p* = .646, *η2* = .004, or effect of condition, *F*(1,58) = .951, *p* = .333, *η2* = .016 were found. There was a significant main effect of group, *F*(1, 58) = 13.778, *p* < .001, *η2* = .192, showing that HA individuals had a higher boundary separation value (*M* = 1.354) compared to LA individuals (*M* = 1.168). Regarding drift rates, a significant interaction was found between group and condition, *F*(1,58) = 7.330, *p* = .009, *η2* = .112 (Supplementary Material File 1: Table S5). Post-hoc comparison using an independent t-test showed significantly higher drift rate values in the HA group compared to LA in the visual correct condition, *t*(58) = 2.795, *p* = .007, *Cohen’s d* = .721, but not in the caption-correct condition, *t*(58) = –1.366, *p* = .177, *Cohen’s d* = –.352. Additionally, there was no main effect of group, *F*(1, 58) = .827, *p* = .367, *η2* = .014; however, a significant main effect of condition was observed, *F*(1, 58) = 4.992, *p* = .029, *η2* = .079, with higher drift rate in the caption-correct condition (*M* = .553) than in the visual-correct condition (*M* = .269; Figure 4). Finally, we found no significant difference between groups regarding duration of non-decisional processes, *t*(58) = 1.376, *p* = .174, *Cohen’s d* = 0.355, and speed of response execution, *t*(58) = 1.547, *p* = .127, *Cohen’s d* = 0.400.

### Association Between Questionnaires and Computational Measures

Correlations between DDM and RL parameters and questionnaires (alexithymia and empathy and their subscales) were assessed (Supplementary Material File 1: Table S6). Boundary separation values in both visual-correct, and caption-correct conditions showed significant positive correlations with total alexithymia score, *r_visual_*(59) = .432, *p_visual_* = .001, *r_caption_*(59) = .389, *p_caption_* = .002, DIF, *r_visual_*(59) = .451, *p_visual_* < .001, *r_caption_*(59) = .414, *p_caption_* = .001, DDF, *r_visual_*(59) = .466, *p_visual_* < .001, *r_caption_*(59) = .379, *p_caption_* = .003, and PD, *r_visual_*(59) = .274, *p_visual_* = .034, *r_caption_*(59) = .355, *p_caption_* = .005. Additionally, positive relationships were found between drift rate values in the visual-correct condition and total alexithymia score, *r*(59) = .340, *p* = .008, DIF, *r*(59) = .334, *p* = .009, DDF, *r*(59) = .382*, p* = .003, PD, *r*(59) = .344*, p* = .007, as well as drift rate values in the caption-correct condition and fantasy, *r*(59) = .298, *p* = .021. On the other hand, caption learning rate was negatively correlated with DIF, *r*(59) = –.321, *p* = .012, and DDF, *r*(59) = –.261, *p* = .044.

## Discussion

Individuals use social cues to infer others’ intentions, emotions, and desires, refining their understanding and responses through a feedback-driven process known as social learning (Schilbach et al., 2013; Zaki et al., 2016). While previous studies have shown that individuals with high alexithymia struggle with non-social learning tasks (Starita et al., 2016; Starita et al., 2019; Starita & Di Pellegrino, 2018), the role of alexithymia in social learning has yet to be investigated. Given the established link between alexithymia and difficulties in social and emotional processing (Borhani et al., 2016; Donges & Suslow, 2017; FeldmanHall et al., 2013; Golbabaei & Borhani, 2024; Grynberg et al., 2010; Grynberg et al., 2012; Grynberg et al., 2018; Lane et al., 1996; Golbabaei, Sammaknejad, et al., 2023; Starita et al., 2018; Van Der Velde et al., 2015), alongside the co-occurrence of this condition in many psychiatric disorders that have been linked to impairment in social interactions (Akinci et al., 2022; Dalili et al., 2015), it is critical to investigate whether this condition impairs social learning. To this end, we examined the social learning process in high alexithymia (HA) and low alexithymia (LA) groups and assessed differences in their learning from visual and caption cues through three different analytical approaches: bin-based analysis, Q-learning, and drift-diffusion modeling.

Our bin-based analysis revealed that individuals with HA showed lower accuracy in the social learning task compared to those with LA, indicating a reduced ability in the HA group to use social cues for inferring others’ emotional states through trial and error. This finding builds on existing literature, demonstrating that individuals with alexithymia not only struggle with learning from non-social stimuli during conditional and instrumental learning (Starita et al., 2016; Starita & Di Pellegrino, 2018), but also exhibit notable deficits in social learning. In line with the predictive coding framework, the observed differences in social learning in alexithymia might relate to challenges in utilizing feedback to form internal representations of social cues (Starita et al., 2016). This could be due to reduced sensitivity to failure (Starita & Di Pellegrino, 2018), altered physiological responses (Borhani et al., 2017; Luminet et al., 2004; Papciak et al., 1985; Stone & Nielson, 2001), or impaired emotional embodiment (Ihme et al., 2014; Scarpazza et al., 2014; Scarpazza et al., 2018). Neuroscientific findings support this view by showing that individuals with high levels of alexithymia exhibit reduced activity in medial frontal cortex (Kano et al., 2011), an important region in adjusting prediction errors (Ridderinkhof et al., 2004), as well as diminished feedback-related negativity, indicating difficulty in internal assessment of predictive errors and updating values based on feedback (Starita et al., 2019). Without constructing these internal representations, individuals may struggle to anticipate future emotional and social events (Starita & Di Pellegrino, 2018). Additionally, deficits in emotional memory could hinder incidental learning and retrieval of emotional information (Suslow et al., 2003), leading to continued exploratory behavior even after acquiring behavior-outcome associations (Ferguson et al., 2009).

Moreover, our correlational analysis, as well as the mixed effects models, not only confirmed the difference in social learning in alexithymia, but also illuminated the specific role of alexithymia facets. Difficulty in identifying feelings (DIF) was associated with lower performance in caption-correct trials, while externally oriented thinking (EOT) was linked to lower performance in visual-correct trials. This is notable because it highlights how distinct alexithymia dimensions contribute to social learning across varied contexts and provides insights into previous research. For instance, while Suslow et al. (2003) reported the negative relationship between DIF and emotional memory for words, Starita et al. (2018) linked EOT to challenges in learning non-verbal cues.

DIF is related to executive functions, including inhibition of unwanted responses and error correction, whereas EOT may reduce salience of social signals, impacting memory consolidation or retrieval (Luminet et al., 2021). Our findings extend this understanding by showing that distinct facets of alexithymia play roles in different social learning contexts: DIF impacts verbal context, while EOT affects visually-based social learning. This implies that alexithymic individuals who struggle to label their own emotions may also find it challenging to encode others’ verbal expression, aligning with evidence suggesting a broader deficit in emotional conceptualization, even at the linguistic level (Hobson et al., 2019; Wotschack & Klann-Delius, 2013). Conversely, EOT is associated with difficulties in mentalizing, a metacognitive process essential for representing one’s inner state and others’ and distinguishing between them (Demers & Koven, 2015; Tuch, 2011). This difficulty leads to a focus on concrete aspects of life and deficiencies in introspection (Demers & Koven, 2015), affective TOM (Demers & Koven, 2015; Pisani et al., 2021), and imaginative fantasy (Sifneos, 1973; Taylor et al., 1991). Consequently, greater engagement with concrete cues likely makes it harder to learn from and disentangle visual information, which may be more complex than verbal information (Lane et al., 1996). The predictive coding framework can help clarify these findings further. DIF may be linked to lower ability in updating priors and reducing the prediction error (reducing entropy) when processing verbal cues, diminishing the HA group’s ability to update beliefs based on feedback. On the contrary, EOT may be associated with an over-weighting of visual sensory input.

Additionally, a positive correlation was found between fantasy and learning ability in the visual-correct condition and overall performance; however, for the visual-correct condition, this effect was reduced to a non-significant trend in the mixed-effects model when including fantasy alongside other subscales and TAS. Higher fantasy is associated with tendencies toward mentalization, context absorption, and creativity (Demers & Koven, 2015; Tuch, 2011), which help individuals link visual cues to target concepts, and infer inner states from feedback, thereby enhancing learning. Interestingly, fantasy has also been linked to learning in the IOWA Gambling Task (Takano et al., 2010). Given that fantasy is typically diminished in individuals with high alexithymia— and is inversely related to EOT (Golbabaei, Barati, et al., 2023; Taylor et al., 1999)—this finding supports our previous results linking EOT to the overall learning performance. Thus, in the context of predictive coding fantasy seems to be necessary for reliably weighting input signals to minimize prediction errors.

It is important to note that the correlational analyses and mixed-effects modeling were used as complementary, rather than competing approaches. Correlations examined direct associations between psychological factors and performance within each condition, whereas mixed-effects models accounted for shared variance among empathy and alexithymia constructs and their subscales. Importantly, mixed-effects models estimate effects relative to unpredicted condition, while correlations assess associations within each condition independently. Thus, mixed-effects results can be interpreted as a more conservative, model-based confirmation of the correlational findings. Notably, the two approaches yielded largely consistent results, reinforcing the robustness of the observed effects.

To further explore the dynamics of social learning, we applied two computational methods, namely reinforcement learning and drift-diffusion modeling. Contrary to our prediction, the Q-learning model revealed no difference in learning rate between the HA and LA groups. However, difficulties in identifying and describing emotions (DIF and DDF) were associated with a lower learning rate for caption-correct targets. This is an intriguing finding, highlighting how distinct facets of alexithymia contribute differently to its behavioral manifestations. Previous research has consistently distinguished these two facets from externally oriented thinking (EOT) dimension (Donges & Suslow, 2017; Goerlich, 2018; Kajanoja et al., 2019; Vanheule, Desmet, Verhaeghe, et al., 2007; Vermeulen, 2021). The recent attention–reappraisal theory of alexithymia (Preece et al., 2017) posits that DIF and DDF are primarily associated with deficits in the reappraisal stage of emotional processing, whereas EOT is linked to early attentional mechanisms, a distinction supported by empirical evidence (Mas & Luminet, 2025). Moreover, higher DIF scores have shown to be associated with lower ability in remembering emotional words in memory tasks (Vermeulen & Luminet, 2009) that could itself influence the learning procedure. In line with this framework, lower engagement in emotional processing observed in individuals high in DIF and DDF (Aaron et al., 2018; Rigby et al., 2020) and having difficulty in memory for emotional words may manifest as reduced value updating within a predictive coding framework.

Despite our findings regarding facets of alexithymia, a potential explanation for the null result of the main analysis is the Q-learning model’s omission of reaction time, which disregards the duration one takes to learn from feedback and make a decision. Since alexithymia is associated with changes in response times, particularly in situations with conflicting information (De Galan et al., 2014; Ihme et al., 2014; Starita & Di Pellegrino, 2018; Zhang et al., 2011), incorporating reaction time and accuracy into the model was essential. Therefore, we utilized drift-diffusion modeling to scrutinize the role of time and accuracy in perceiving, interpreting, and representing social information.

The drift diffusion model yielded notable findings. The HA group showed a higher boundary separation parameter, indicating they require more information before making a decision. This conservatism suggests a greater need for evidence to infer others’ emotions in the conflict-ridden situations of our task, likely affecting how they learn from prediction errors, expected values, and feedback in favor of particular social cues. This is also in line with previous literature showing the slower processing of emotional information in alexithymia (Ihme et al., 2014; Starita et al., 2018). Importantly, conservatism did not lead to more accurate decisions and better social learning in alexithymia. Furthermore, boundary separation correlated with total TAS-20 score and its affective subcomponents—DIF and DDF—indicating that this need for additional evidence is aspect-specific, involving the affective components of alexithymia rather than the cognitive component. Moreover, this aligns with previous findings indicating that individuals with high alexithymia require more emotional intensity to recognize emotions (Starita et al., 2018). Intriguingly, higher personal distress was linked to more conservative information accumulation across conditions. At elevated levels, personal distress often associated with alexithymia (Banzhaf et al., 2018; Beadle & McCormick, 2014; Golbabaei, Barati, et al., 2023; Nam et al., 2020), can lead to avoidance rather than perspective-taking (Davis, 1983; Kim & Han, 2018; Zaki, 2014, 2020).

Additionally, our results revealed a significant interaction between the group and both the starting point (indicative of bias) and drift rate (a measure of information accumulation). In the visual-correct condition, the HA group showed significantly higher values for both parameters, and they were non-significantly lower in the caption-correct condition. This finding suggests that individuals with HA rely more on visual cues for value updates, displaying stronger perceptual bias and faster accumulation of information for visual cues over verbal ones. However, given that their overall accuracy was lower, it seems their need for perceptual information and conservativeness outweighed the benefits of perceptual bias and information accumulation speed. The higher starting point observed in the visual condition for the HA group may reflect a bias toward relying on visual cues when inferring emotional information. This bias could arise from their difficulties in processing facial emotions and may represent a compensatory mechanism. By contrast, in the caption condition, semantic information was more accessible, which facilitated processing and learning, although participants’ learning in this condition was still not entirely accurate. From a predictive coding lens (Friston, 2005; Shaw et al., 2025), this bias in relying on visual cues can be due to placing greater weight on visual priors rather than verbal ones. While this can be an interesting and important issue, we cannot make such inference directly from our results, and it should be examined by Bayesian models in the future.

Importantly, participants generally exhibited a higher drift rate, or speed of information accumulation, in the visual-correct condition, indicating a prioritization of visual information even though it served as the main cue in only half of the trials. Consequently, the HA group appeared to rely more heavily on the prominent visual cues rather than adjusting their information accumulation in response to feedback. This further indicates that individuals with high levels of alexithymia not only assign greater weight to visual priors but also update them more rapidly, thereby minimizing prediction errors more quickly. Overall, the present findings indicate that alexithymia is associated with difficulties in social learning. This is an important observation, as it suggests that impairments in processing social information observed in psychiatric disorders, where alexithymia is highly prevalent, may be at least partly attributable to alexithymia itself.

In addition, our findings have the potential to suggest that difficulties in social learning may contribute to maladaptive social cognitive abilities, such as impaired empathy (Alkan Härtwig et al., 2020; Moriguchi et al., 2007), in individuals with high levels of alexithymia. In line with the predictive coding framework, computational models of empathy suggest that these findings may reflect difficulties in efficiently accumulating social evidence to infer others’ emotional states. As a result, individuals with HA may be less able to use feedback and prediction errors to update the value of their empathic inferences. Based on this model, their perceptual bias toward visual cues indicates that they tend to hold a greater engagement with concrete cues rather than integrating multiple cues to update their inferred empathy.

Despite these promising findings, our study has limitations and areas for improvement. Consistent with previous studies (Berenbaum & Irvin, 1996; Borhani et al., 2016; Borhani et al., 2017; Gaggero et al., 2022; Starita & di Pellegrino, 2018; Vanheule, Desmet, Meganck, et al., 2007; Yu et al., 2024), we adopted a threshold-based grouping into high- and low-alexithymia cohorts. However, this design choice substantially constrained recruitment (eligible participants are scarce near the thresholds), resulting in a sample size smaller than ideal and reduced power for between-group contrasts. The verbal feedback in our study was presented as text, potentially reducing its intensity compared to the visual cues, which were faces. Future studies might consider using auditory cues for verbal feedback, enhancing ecological validity and the intensity comparability with visual cues. Additionally, whereas our trials presented conflicting visual and caption cues following Zaki et al. (2016), future research could incorporate trials with congruent or single cues to investigate group differences when no conflict exists. While our focus was on social learning at the behavioral level, future studies should explore the neural substrates underlying social learning in individuals with high alexithymia (HA), given suggested overlaps (Zaki et al., 2016) with the neural circuits for emotional processing. Another important consideration is our use of fast-dm and classical drift–diffusion modeling. These approaches assume independence across participants and fit each individual separately (Voss et al., 2015; Voss & Voss, 2007), which can limit power to detect between-group effects. In contrast, hierarchical Bayesian DDMs (HDDMs; Jiang, 2022; Lee & Wagenmakers, 2014; Vandekerckhove et al., 2011; Wiecki et al., 2013) estimate group- and subject-level parameters jointly with partial pooling, stabilizing noisy individual estimates and increasing sensitivity to group differences. It boosts power to detect theorized group differences, while stabilizing the remaining parameters through hierarchical pooling. HDDMs also provide sharper uncertainty quantification by yielding full posterior distributions for parameters at each level. In addition, they flexibly incorporate trial-level regressors and random effects, and support posterior predictive checks to assess absolute fit to full RT distributions. Given our limited sample size, these advantages may help explain the absence of reliable group differences in the drift–starting-point interaction (beyond the observed group × condition effect). We therefore recommend that future work employ HDDMs to more powerfully test theory-driven group effects while allowing other parameters to be informed by the joint sample.

In conclusion, this study suggests that alexithymia significantly impacts social learning, with individuals with HA displaying lower accuracy when learning from social feedback cues. Our findings underscore the pivotal role of time in differentiating social learning processes between HA and LA groups. While individuals with HA required more evidence accumulation to infer emotions, this increased demand may contribute to impaired social learning. Conversely, they exhibited faster evidence accumulation and a perceptual bias favoring visual cues, perhaps relying on more salient evidence or employing compensatory strategies. These insights into social learning deficits in alexithymia may shed light on the broader challenges in social cognition associated with this condition.

## Data Accessibility Statement

The codes used for this study have now been uploaded to an open-access repository https://osf.io/rh4g8/overview. All results supporting the findings of this study are included in the manuscript and in the supplementary materials (Supplementary Material Files 1–4).

## Additional Files

The additional files for this article can be found as follows:

10.5334/cpsy.153.s1 Supplementary Material File 1. Detailed ANOVA results for group and condition effects, and correlations with questionnaire measures.

10.5334/cpsy.153.s2 Supplementary Material File 2. Trial-level analyses conducted using mixed-effects models.

10.5334/cpsy.153.s3 Supplementary Material File 3. Detailed mixed-effects model results including covariates.

10.5334/cpsy.153.s4 Supplementary Material File 4. Detailed results from reinforcement learning model analyses.

## Third Party Material

All of the material is owned by the authors, and no permissions are required.
