# Supplementary material for: The Role of Alexithymia in Social Learning and Feedback-Driven Social Inferences
Source: Comput Psychiatr. 2026 Mar 19;10(1):35–57. doi: 10.5334/cpsy.153 (PMC13004067; doi:10.5334/cpsy.153)
Supplement: Supplementary Material File 1. — Detailed ANOVA results for group and condition effects, and correlations with questionnaire measures. [file cpsy-10-1-153-s1.pdf]

Supplementary Material 1

Social targets	Video	*ICC*		*95% Confidence Interval*		*F*		*p*
		Single	Average	Lower Bound	Upper Bound	*df 1*	*df 2*	
A (Male)	Happy	.726	.981	.520	.918	7	133	<.001
	Sad	.719	.981	.511	.916	7	133	<.001
B (Female)	Happy	.710	.980	.500	.912	7	133	<.001
	Sad	.695	.978	.481	.949	7	133	<.001
C (Female)	Happy	.777	.986	.589	.937	7	133	<.001
	Sad	.561	.962	.336	.846	7	133	<.001

Table S1 Inter-rater Consistency for Emotion Ratings.

Note: Single measure ICC refers to the reliability (or consistency) of one single measurement or one rater's score. Average measure ICC refers: The reliability of the average of multiple raters or measurements.

Table S2 Means, Standard Deviations, and t-test Results for Pairwise Comparisons of Learning Accuracy in Interaction Between Conditions and Blocks.

Condition	Block	*M*	*SD*	*t*	*p*	*Cohen's d*
Visual-correct	One - Two	-.950	2.639	-2.789	.007	-.360
	One - Three	-1.533	2.752	-4.315	< .001	-.557
	One - Four	-2.017	2.819	-5.541	< .001	-.715
	Two - Three	-.583	2.173	-2.079	.042	-.268
	Two - Four	-1.067	2.510	-3.292	.002	-.425
	Three - Four	-.483	2.332	-1.605	.114	-.207
Caption-correct	One - Two	-1.000	2.810	-2.756	.008	-.356
	One - Three	-1.417	3.381	-3.245	.002	-.419
	One - Four	-2.050	3.170	-5.009	< .001	-.647
	Two - Three	-.417	2.513	-1.284	.204	-.166
	Two - Four	-1.050	2.062	-3.944	< .001	-.509
	Three - Four	-.633	2.058	-2.384	.020	-.308
Unpredicted	One - Two	-.233	2.513	-.719	.475	-.093
	One - Three	.017	2.274	.057	.955	.007
	One - Four	-.100	2.516	-.308	.759	-.040
	Two - Three	.250	2.282	.849	.400	.110
	Two - Four	.133	2.404	.430	.669	.055
	Three - Four	-.117	2.351	-.384	.702	-.050

Table S3 Means, Standard Deviations, and t-test Results for Pairwise Comparisons of Learning Accuracy in Blocks.

Blocks	*M*	*SD*	*t*	*p*	*Cohen's d*
One – Two	-2.183	4.966	-3.406	.001	-.440
One – Three	-2.933	5.095	-4.459	< .001	-.576
One – Four	-4.167	5.397	-5.980	< .001	-.772
Two – Three	-.750	3.843	-1.512	.136	-.195
Two – Four	-1.983	4.440	-3.460	.001	-.447
Three – Four	-1.233	4.276	-2.234	.029	-.288

Table S4 Correlations Between TAS-20, IRI Scores, and Correct Responses in Visual-Correct, Caption-Correct, and All Conditions.

Measure	Number of correct responses in visual-correct condition		Number of correct responses in caption-correct condition		Total number of correct responses	
	r	*p*	*r*	*p*	*r*	*p*
TAS-20	-.195	.135	-.239	.066	-.297*	.031
DIF	-.144	.273	-.294*	.023	-.287*	.029
DDF	-.132	.316	-.171	.192	-.197	.132
EOT	-.292*	.023	-.147	.262	-.269*	.038
IRI	.234	.073	.059	.655	.230	.078
EC	.111	.397	.103	.432	.158	.228
PT	.105	.423	.193	.139	.239	.066
PD	.022	.866	-.241	.063	-.140	.287
FS	.275*	.034	.128	.329	.292*	.024

* = $p < .05$.

Table S5 ANOVA Results for the Drift-Diffusion Modeling Analysis.

Measure	Relative starting point				Boundary separation				Drift rate			
	df	*F*	*p*	*η2*	*df*	*F*	*p*	*η2*	*df*	*F*	*p*	*η2*
Group	1, 58	.422	.518	.007	1, 58	13.778	< .001	.192	1, 58	0.827	.367	.014
Condition	1, 58	1.390	.243	.023	1, 58	.951	.333	.016	1, 58	4.492	.029	.079
Condition*Group	1, 58	7.559	.008	.115	1, 58	.214	.646	.004	1, 58	7.330	.009	.112

Table S6 Correlation Between TAS-20, IRI Scores, and DDM and RL Parameters.

Measure	Relative starting point		Threshold separation		Drift rate		Learning rate	
	Visual	Caption	Visual	Caption	Visual	Caption	Visual	Caption
TAS-20	.237	-.155	.432**	.389**	.340**	-.191	-.010	-.237
DIF	.234	-.157	.451**	.414**	.334**	-.190	-.050	-.321*
DDF	.209	-.181	.466**	.379**	.382**	-.099	-.156	-.261*
EOT	.190	-.046	.177	.204	.143	-.254	-.064	.035
IRI	.016	-.052	.093	.235	.196	.180	-.222	-.199
EC	.097	.007	-.090	.096	.102	.023	-.132	.034
PT	-.008	.231	-.222	-.089	-.030	.176	-.076	-.113
PD	-.058	-.222	.274*	.355**	.344**	-.094	-.213	-.154
FS	.016	-.056	.142	.113	.003	.298*	-.082	-.191

* = p < .05; ** = p < 0.01.
