# Supplementary material for: The Role of Alexithymia in Social Learning and Feedback-Driven Social Inferences
Source: Comput Psychiatr. 2026 Mar 19;10(1):35–57. doi: 10.5334/cpsy.153 (PMC13004067; doi:10.5334/cpsy.153)
Supplement: Supplementary Material File 2. — Trial-level analyses conducted using mixed-effects models. [file cpsy-10-1-153-s2.pdf]

We examined the effects of Group (low vs. high), Condition (visual-correct, caption/verbal-correct, unpredicted), and learning using mixed effects models. Learning can be operationalized as performance change across blocks or across trials. The performance change across blocks is presented in the main text, using repeated measures ANOVA, thus here, we ran a logistic mixed effects model on trial-level accuracy. Because the model includes interactions, main effects are evaluated at the reference levels of the other factors. We set unpredicted as the reference for Condition, and low alexithymia as the reference level for Group, so main-effect estimates are interpreted for the unpredicted condition and the low alexithymia group. To avoid conflating this with Group, we first fit models without Group and then added it.

Consistent with the Mixed ANOVA reported in the main text, trial-level modeling showed significant Condition × learning interactions for the caption- and visual-correct conditions (Table S1), indicating performance improved over trials in both conditions. We also observed significant Group × Condition interactions for the caption-correct and visual-correct conditions (Table S2), with the low-alexithymia group outperforming the high-alexithymia group in both.

Table S1 Linear Mixed-Effects Logistic Regression Analysis of Correct Responses as a Function of Condition and Trial Number.

Measure	*Estimate*	*Std. Error*	*z value*	*Pr(>|z|)*
(Intercept)	-.022	.065	-.340	.734
Trial	-.017	.038	-.444	.657
ConditionVerbal	.770	.056	13.795	< .001
ConditionVisual	.860	.056	15.239	< .001
Trial:ConditionVerbal	.292	.056	5.208	< .001
Trial:ConditionVisual	.331	.057	5.847	< .001

Table S2 Linear Mixed-Effects Logistic Regression Analysis of Correct Responses as a Function of Condition, Trial Number, and Group.

Measure	*Estimate*	*Std. Error*	*z value*	*Pr(>|z|)*
(Intercept)	-.036	.089	-.41	.68193
Trial	.000	.053	.003	.99787
ConditionVerbal	1.024	.081	12.618	< .001
ConditionVisual	1.034	.081	12.752	< .001
GroupHigh	.030	.127	.236	.813
Trial:ConditionVerbal	.330	.0811	4.069	< .001
Trial:ConditionVisual	.316	.082	3.868	< .001
Trial:GroupHigh	-.038	.076	-.5	.617
ConditionVerbal:GroupHigh	-.491	.112	-4.384	< .001
ConditionVisual:GroupHigh	-.345	.113	-3.063	.002
Trial:ConditionVerbal:GroupHigh	-.061	.113	-.539	.589
Trial:ConditionVisual:GroupHigh	.034	.113	.301	.763
