# Supplementary material for: The Role of Alexithymia in Social Learning and Feedback-Driven Social Inferences
Source: Comput Psychiatr. 2026 Mar 19;10(1):35–57. doi: 10.5334/cpsy.153 (PMC13004067; doi:10.5334/cpsy.153)
Supplement: Supplementary Material File 3. — Detailed mixed-effects model results including covariates. [file cpsy-10-1-153-s3.pdf]

Given the overlap between TAS-20 and empathy (IRI), we complemented the correlational analyses in the main text with mixed effects models that allow us to include Condition and Time (learning) explicitly. Learning was modeled as continuous change either across blocks or across trials. Accordingly, we ran two specifications: (i) a linear mixed effects model on block-wise percent correct, and (ii) a logistic mixed effects model on trial-wise accuracy.

We proceeded in stages. First, we fit models with TAS-20, Condition, and Time (Block/TrialNo) and their relevant interactions (Tables S1-2). Second, we added IRI total score to these models (Tables S3-4). Third, we included TAS-20 alongside the IRI subscales in a single model (Tables S5–6). Because subscales share variance, we then fit separate models pairing TAS-20 with each IRI subscale (Tables S7–14). Finally, we fit a model including the three TAS-20 subscales together (Tables S15–16).

The Questionnaire × Condition term is the mixed-effects analogue of the condition-wise correlations reported in the main text. The Questionnaire × Condition × Time term tests whether questionnaire scores moderate learning within a condition. Note that learning need not be linear, so a non-significant three-way interaction does not preclude learning effects.

Table S1 Mixed-Effects Regression Analysis of Correct Responses as a Function of Condition, Block Number, and TAS-20 Score.

Measure	*Estimate*	*Std. Error*	*t value*	*Pr(>|t|)*
(Intercept)	-.547	.069	-7.967	.000
ConditionCaption	.780	.075	10.371	.000
ConditionVisual	.861	.075	11.455	.000
Block	.002	.053	.038	.970
TAS	.010	.069	.149	.882
ConditionCaption:Block	.264	.075	3.508	.000
ConditionVisual:Block	.267	.075	3.544	.000
ConditionCaption:TAS	-.234	.075	-3.105	.002
ConditionVisual:TAS	-.150	.075	-1.996	.046
Block:TAS	-.014	.053	-.270	.787
ConditionCaption:Block:TAS	-.027	.075	-.352	.725
ConditionVisual:Block:TAS	.023	.075	.311	.756

Table S2 Linear Mixed-Effects Logistic Regression Analysis of Correct Responses as a Function of Condition, Trial Number, and TAS-20 Score.

Measure	*Estimate*	*Std. Error*	*z value*	*Pr(>|z|)*
(Intercept)	-.022	.063	-.344	.731
ConditionCaption	.781	.056	13.910	.000
ConditionVisual	.861	.056	15.250	.000
Trial	-.019	.038	-.488	.626
TAS	.018	.063	.284	.776
ConditionCaption:Trial	.302	.056	5.352	.000
ConditionVisual:Trial	.333	.057	5.868	.000
ConditionCaption:TAS	-.277	.056	-4.939	.000
ConditionVisual:TAS	-.157	.056	-2.784	.005
Trial:TAS	-.017	.038	-.446	.656
ConditionCaption:Trial:TAS	-.039	.056	-.683	.495
ConditionVisual:Trial:TAS	.014	.057	.248	.804

Table S3 Mixed-Effects Regression Analysis of Correct Responses as a Function of Condition, Block Number, TAS-20, and IRI Score.

Measure	*Estimate*	*Std. Error*	*t value*	*Pr(>|t|)*
(Intercept)	-.547	.067	-8.141	.000
ConditionCaption	.780	.075	10.370	.000
ConditionVisual	.861	.075	11.453	.000
Block	.002	.053	.038	.970
TAS	-.002	.068	-.029	.977
IRI	.070	.068	1.021	.309
ConditionCaption:Block	.264	.075	3.507	.000
ConditionVisual:Block	.267	.075	3.543	.000
ConditionCaption:TAS	-.238	.076	-3.120	.002
ConditionVisual:TAS	-.172	.076	-2.256	.024
ConditionCaption:IRI	.028	.076	.360	.719
ConditionVisual:IRI	.127	.076	1.663	.097
Block:TAS	-.019	.054	-.358	.720
Block:IRI	.028	.054	.527	.599
ConditionCaption:Block:TAS	-.032	.076	-.414	.679
ConditionVisual:Block:TAS	.023	.076	.301	.763
ConditionCaption:Block:IRI	.029	.076	.384	.701
ConditionVisual:Block:IRI	.002	.076	.028	.978

Table S4 Linear Mixed-Effects Logistic Regression Analysis of Correct Responses as a Function of Condition, Trial Number, TAS-20, and IRI Score.

Measure	*Estimate*	*Std. Error*	*z value*	*Pr(>|z|)*
(Intercept)	-.022	.061	-.357	.721
ConditionCaption	.782	.056	13.920	.000
ConditionVisual	.869	.057	15.335	.000
Trial	-.018	.038	-.476	.634
TAS	.005	.062	.082	.934
IRI	.077	.062	1.235	.217
ConditionCaption:Trial	.303	.056	5.376	.000
ConditionVisual:Trial	.338	.057	5.939	.000
ConditionCaption:TAS	-.289	.057	-5.035	.000
ConditionVisual:TAS	-.191	.058	-3.306	.001
ConditionCaption:IRI	.048	.057	.839	.402
ConditionVisual:IRI	.155	.058	2.682	.007
Trial:TAS	-.022	.039	-.574	.566
Trial:IRI	.023	.038	.601	.548
ConditionCaption:Trial:TAS	-.044	.058	-.768	.442
ConditionVisual:Trial:TAS	.005	.058	.090	.928
ConditionCaption:Trial:IRI	.023	.057	.404	.686
ConditionVisual:Trial:IRI	.034	.058	.598	.550

Table S5 Mixed-Effects Regression Analysis of Correct Responses as a Function of Condition, Block Number, TAS-20, and IRI Subscales Score.

Measure	*Estimate*	*Std. Error*	*t value*	*Pr(>|t|)*
(Intercept)	-.547	.067	-8.110	.000
ConditionCaption	.780	.075	10.387	.000
ConditionVisual	.861	.075	11.471	.000
Block	.002	.053	.038	.970
TAS	.005	.080	.062	.951
EC	.007	.070	.094	.925
PT	.047	.072	.662	.509
FS	.039	.071	.551	.582
PD	.034	.080	.418	.677
ConditionCaption:Block	.264	.075	3.513	.000
ConditionVisual:Block	.267	.075	3.549	.000
ConditionCaption:TAS	-.119	.089	-1.333	.183
ConditionVisual:TAS	-.187	.089	-2.096	.036
ConditionCaption:EC	.082	.078	1.061	.289
ConditionVisual:EC	.031	.078	.405	.686
ConditionCaption:PT	.042	.080	.529	.597
ConditionVisual:PT	-.038	.080	-.478	.633
ConditionCaption:FS	.071	.079	.900	.368
ConditionVisual:FS	.135	.079	1.708	.088
ConditionCaption:PD	-.210	.089	-2.345	.019
ConditionVisual:PD	.048	.089	.538	.591
Block:TAS	-.007	.063	-.104	.917
Block:EC	.040	.055	.731	.465
Block:PT	.018	.056	.316	.752
Block:FS	.001	.056	.017	.986
Block:PD	-.010	.063	-.153	.878
ConditionCaption:Block:TAS	-.004	.089	-.046	.964
ConditionVisual:Block:TAS	.025	.089	.282	.778
ConditionCaption:Block:EC	-.043	.078	-.556	.579
ConditionVisual:Block:EC	-.068	.078	-.872	.384
ConditionCaption:Block:PT	-.003	.080	-.041	.968
ConditionVisual:Block:PT	-.004	.080	-.047	.962
ConditionCaption:Block:FS	.100	.079	1.266	.206
ConditionVisual:Block:FS	.059	.079	.744	.457
ConditionCaption:Block:PD	-.043	.090	-.476	.634
ConditionVisual:Block:PD	.000	.090	.001	.999

Table S6 Linear Mixed-Effects Logistic Regression Analysis of Correct Responses as a Function of Condition, Trial Number, TAS-20, and IRI Subscales Score.

Measure	*Estimate*	*Std. Error*	*z value*	*Pr(>|z|)*
(Intercept)	-.022	.060	-.357	.721
ConditionCaption	.796	.057	14.048	.000
ConditionVisual	.870	.057	15.337	.000
Trial	-.019	.038	-.492	.623
TAS	.015	.072	.212	.832
EC	.006	.062	.100	.920
PT	.072	.064	1.131	.258
FS	.028	.063	.449	.654
PD	.042	.072	.588	.556
ConditionCaption:Trial	.317	.057	5.568	.000
ConditionVisual:Trial	.340	.057	5.974	.000
ConditionCaption:TAS	-.147	.067	-2.195	.028
ConditionVisual:TAS	-.220	.068	-3.222	.001
ConditionCaption:EC	.073	.058	1.253	.210
ConditionVisual:EC	.024	.059	.415	.678
ConditionCaption:PT	.053	.060	.879	.380
ConditionVisual:PT	-.050	.060	-.827	.408
ConditionCaption:FS	.119	.059	2.011	.044
ConditionVisual:FS	.169	.059	2.838	.005
ConditionCaption:PD	-.244	.068	-3.614	.000
ConditionVisual:PD	.079	.068	1.162	.245
Trial:TAS	-.010	.045	-.228	.819
Trial:EC	.002	.039	.063	.950
Trial:PT	.012	.040	.291	.771
Trial:FS	.030	.040	.755	.450
Trial:PD	-.013	.045	-.279	.781
ConditionCaption:Trial:TAS	-.013	.067	-.197	.844
ConditionVisual:Trial:TAS	-.001	.068	-.015	.988
ConditionCaption:Trial:EC	-.028	.059	-.477	.633
ConditionVisual:Trial:EC	-.016	.059	-.274	.784
ConditionCaption:Trial:PT	-.003	.060	-.054	.957
ConditionVisual:Trial:PT	.019	.061	.312	.755
ConditionCaption:Trial:FS	.087	.060	1.458	.145
ConditionVisual:Trial:FS	.026	.060	.438	.662
ConditionCaption:Trial:PD	-.058	.068	-.858	.391
ConditionVisual:Trial:PD	.037	.068	.536	.592

Table S7 Mixed-Effects Regression Analysis of Correct Responses as a Function of Condition, Block Number, TAS-20, and Empathic Concern Score.

Measure	*Estimate*	*Std. Error*	*t value*	*Pr(>|t|)*
(Intercept)	-.547	.068	-7.995	.000
ConditionCaption	.780	.075	10.348	.000
ConditionVisual	.861	.075	11.429	.000
Block	.002	.053	.038	.970
TAS	.009	.069	.134	.894
EC	.023	.069	.340	.734
ConditionCaption:Block	.264	.075	3.500	.000
ConditionVisual:Block	.267	.075	3.536	.000
ConditionCaption:TAS	-.238	.075	-3.147	.002
ConditionVisual:TAS	-.153	.075	-2.028	.043
ConditionCaption:EC	.084	.075	1.116	.265
ConditionVisual:EC	.063	.075	.830	.407
Block:TAS	-.016	.053	-.305	.760
Block:EC	.041	.053	.773	.440
ConditionCaption:Block:TAS	-.025	.076	-.336	.737
ConditionVisual:Block:TAS	.026	.076	.344	.731
ConditionCaption:Block:EC	-.024	.076	-.323	.747
ConditionVisual:Block:EC	-.055	.076	-.723	.470

Table S8 Linear Mixed-Effects Logistic Regression Analysis of Correct Responses as a Function of Condition, Trial Number, TAS-20, and Empathic Concern Score.

Measure	*Estimate*	*Std. Error*	*z value*	*Pr(>|z|)*
(Intercept)	-.022	.063	-.347	.728
ConditionCaption	.783	.056	13.925	.000
ConditionVisual	.861	.056	15.300	.000
Trial	-.018	.038	-.483	.629
TAS	.017	.063	.272	.786
EC	.024	.063	.385	.700
ConditionCaption:Trial	.302	.056	5.351	.000
ConditionVisual:Trial	.333	.057	5.872	.000
ConditionCaption:TAS	-.283	.056	-5.021	.000
ConditionVisual:TAS	-.161	.057	-2.843	.004
ConditionCaption:EC	.091	.056	1.614	.107
ConditionVisual:EC	.063	.057	1.111	.266
Trial:TAS	-.019	.038	-.492	.622
Trial:EC	.009	.038	.229	.819
ConditionCaption:Trial:TAS	-.039	.057	-.685	.494
ConditionVisual:Trial:TAS	.015	.057	.267	.790
ConditionCaption:Trial:EC	-.013	.057	-.224	.823
ConditionVisual:Trial:EC	-.002	.057	-.040	.968

Table S9 Mixed-Effects Regression Analysis of Correct Responses as a Function of Condition, Block Number, TAS-20, and Perspective Taking Score.

Measure	*Estimate*	*Std. Error*	*t value*	*Pr(>|t|)*
(Intercept)	-.547	.068	-7.996	.000
ConditionCaption	.780	.075	10.350	.000
ConditionVisual	.861	.075	11.431	.000
Block	.002	.053	.038	.970
TAS	.024	.071	.343	.732
PT	.053	.071	.753	.453
ConditionCaption:Block	.264	.075	3.501	.000
ConditionVisual:Block	.267	.075	3.537	.000
ConditionCaption:TAS	-.213	.078	-2.727	.007
ConditionVisual:TAS	-.153	.078	-1.964	.050
ConditionCaption:PT	.078	.078	.997	.319
ConditionVisual:PT	-.012	.078	-.159	.874
Block:TAS	-.008	.055	-.150	.881
Block:PT	.023	.055	.416	.677
ConditionCaption:Block:TAS	-.023	.078	-.293	.769
ConditionVisual:Block:TAS	.023	.078	.299	.765
ConditionCaption:Block:PT	.014	.078	.173	.863
ConditionVisual:Block:PT	.000	.078	-.001	.999

Table S10 Linear Mixed-Effects Logistic Regression Analysis of Correct Responses as a Function of Condition, Trial Number, TAS-20, and Perspective Taking Score.

Measure	*Estimate*	*Std. Error*	*z value*	*Pr(>|z|)*
(Intercept)	-.022	.062	-.356	.722
ConditionCaption	.786	.056	13.954	.000
ConditionVisual	.861	.056	15.248	.000
Trial	-.018	.038	-.482	.630
TAS	.038	.064	.588	.556
PT	.076	.064	1.177	.239
ConditionCaption:Trial	.306	.057	5.408	.000
ConditionVisual:Trial	.333	.057	5.884	.000
ConditionCaption:TAS	-.257	.058	-4.436	.000
ConditionVisual:TAS	-.161	.058	-2.759	.006
ConditionCaption:PT	.091	.059	1.545	.122
ConditionVisual:PT	-.017	.058	-.297	.767
Trial:TAS	-.012	.039	-.310	.757
Trial:PT	.019	.039	.488	.626
ConditionCaption:Trial:TAS	-.039	.058	-.678	.498
ConditionVisual:Trial:TAS	.021	.059	.351	.726
ConditionCaption:Trial:PT	.007	.059	.124	.901
ConditionVisual:Trial:PT	.024	.059	.415	.678

Table S11 Mixed-Effects Regression Analysis of Correct Responses as a Function of Condition, Block Number, TAS-20, and Fantasy Score.

Measure	*Estimate*	*Std. Error*	*t value*	*Pr(>|t|)*
(Intercept)	-.547	.067	-8.170	.000
ConditionCaption	.780	.075	10.395	.000
ConditionVisual	.861	.075	11.500	.000
Block	.002	.053	.038	.970
TAS	.010	.067	.148	.882
FS	.054	.067	.802	.424
ConditionCaption:Block	.264	.075	3.516	.000
ConditionVisual:Block	.267	.075	3.550	.000
ConditionCaption:TAS	-.234	.075	-3.120	.002
ConditionVisual:TAS	-.151	.075	-2.010	.045
ConditionCaption:FS	.068	.075	.903	.367
ConditionVisual:FS	.143	.075	1.900	.058
Block:TAS	-.014	.053	-.272	.786
Block:FS	.012	.053	.223	.824
ConditionCaption:Block:TAS	-.027	.075	-.360	.719
ConditionVisual:Block:TAS	.023	.075	.308	.758
ConditionCaption:Block:FS	.084	.075	1.118	.264
ConditionVisual:Block:FS	.043	.075	.572	.568

Table S12 Linear Mixed-Effects Logistic Regression Analysis of Correct Responses as a Function of Condition, Trial Number, TAS-20, and Fantasy Score.

Measure	*Estimate*	*Std. Error*	*z value*	*Pr(>|z|)*
(Intercept)	-.021	.061	-.350	.727
ConditionCaption	.786	.056	13.956	.000
ConditionVisual	.868	.057	15.318	.000
Trial	-.019	.038	-.490	.624
TAS	.018	.061	.298	.766
FS	.049	.061	.796	.426
ConditionCaption:Trial	.308	.057	5.443	.000
ConditionVisual:Trial	.337	.057	5.930	.000
ConditionCaption:TAS	-.283	.056	-5.029	.000
ConditionVisual:TAS	-.163	.057	-2.870	.004
ConditionCaption:FS	.106	.056	1.903	.057
ConditionVisual:FS	.176	.056	3.131	.002
Trial:TAS	-.019	.038	-.492	.622
Trial:FS	.031	.038	.814	.416
ConditionCaption:Trial:TAS	-.043	.057	-.758	.448
ConditionVisual:Trial:TAS	.013	.057	.228	.820
ConditionCaption:Trial:FS	.067	.056	1.192	.233
ConditionVisual:Trial:FS	.029	.056	.512	.609

Table S13 Mixed-Effects Regression Analysis of Correct Responses as a Function of Condition, Block Number, TAS-20, and Personal Distress Score.

Measure	*Estimate*	*Std. Error*	*t value*	*Pr(>|t|)*
(Intercept)	-.547	.069	-7.940	.000
ConditionCaption	.780	.075	10.400	.000
ConditionVisual	.861	.075	11.500	.000
Block	.002	.053	.038	.970
TAS	-.009	.080	-.116	.908
PD	.038	.080	.474	.636
ConditionCaption:Block	.264	.075	3.520	.000
ConditionVisual:Block	.267	.075	3.560	.000
ConditionCaption:TAS	-.136	.087	-1.560	.119
ConditionVisual:TAS	-.192	.087	-2.200	.028
ConditionCaption:PD	-.190	.087	-2.170	.030
ConditionVisual:PD	.081	.087	.927	.354
Block:TAS	-.011	.062	-.182	.856
Block:PD	-.006	.062	-.099	.921
ConditionCaption:Block:TAS	-.012	.087	-.137	.891
ConditionVisual:Block:TAS	.022	.087	.247	.805
ConditionCaption:Block:PD	-.028	.087	-.324	.746
ConditionVisual:Block:PD	.004	.087	.041	.967

Table S14 Linear Mixed-Effects Logistic Regression Analysis of Correct Responses as a Function of Condition, Trial Number, TAS-20, and Personal Distress Score.

Measure	*Estimate*	*Std. Error*	*z value*	*Pr(>|z|)*
(Intercept)	-.022	.063	-.343	.731
ConditionCaption	.785	.056	13.946	.000
ConditionVisual	.865	.057	15.297	.000
Trial	-.018	.038	-.486	.627
TAS	-.004	.074	-.053	.958
PD	.042	.074	.574	.566
ConditionCaption:Trial	.307	.057	5.420	.000
ConditionVisual:Trial	.336	.057	5.915	.000
ConditionCaption:TAS	-.165	.066	-2.518	.012
ConditionVisual:TAS	-.224	.067	-3.331	.001
ConditionCaption:PD	-.214	.066	-3.237	.001
ConditionVisual:PD	.120	.067	1.784	.074
Trial:TAS	-.014	.044	-.322	.748
Trial:PD	-.005	.044	-.112	.911
ConditionCaption:Trial:TAS	-.014	.066	-.216	.829
ConditionVisual:Trial:TAS	-.006	.067	-.088	.930
ConditionCaption:Trial:PD	-.042	.066	-.634	.526
ConditionVisual:Trial:PD	.033	.067	.497	.619

Table S15 Mixed-Effects Regression Analysis of Correct Responses as a Function of Condition, Block Number, and TAS-20 Subscales.

Measure	*Estimate*	*Std. Error*	*t value*	*Pr(>|t|)*
(Intercept)	-.547	.068	-7.997	.000
ConditionCaption	.780	.074	10.483	.000
ConditionVisual	.861	.074	11.578	.000
Block	.002	.053	.038	.969
DIF	.047	.150	.312	.756
DDF	-.034	.140	-.245	.806
EOT	-.004	.086	-.044	.965
ConditionCaption:Block	.264	.074	3.545	.000
ConditionVisual:Block	.267	.074	3.582	.000
ConditionCaption:DIF	-.650	.163	-3.982	.000
ConditionVisual:DIF	.015	.163	.089	.929
ConditionCaption:DDF	.372	.153	2.440	.015
ConditionVisual:DDF	.014	.153	.089	.929
ConditionCaption:EOT	.058	.094	.615	.539
ConditionVisual:EOT	-.235	.094	-2.501	.013
Block:DIF	.011	.116	.096	.923
Block:DDF	-.024	.108	-.220	.826
Block:EOT	-.003	.066	-.053	.958
ConditionCaption:Block:DIF	-.027	.163	-.164	.870
ConditionVisual:Block:DIF	.007	.163	.043	.965
ConditionCaption:Block:DDF	.028	.153	.184	.854
ConditionVisual:Block:DDF	.008	.153	.051	.960
ConditionCaption:Block:EOT	-.036	.094	-.379	.705
ConditionVisual:Block:EOT	.014	.094	.145	.885

Table S16 Linear Mixed-Effects Logistic Regression Analysis of Correct Responses as a Function of Condition, Trial Number, and TAS-20 Subscales.

Measure	*Estimate*	*Std. Error*	*z value*	*Pr(>|z|)*
(Intercept)	-.022	.063	-.345	.730
ConditionCaption	.795	.057	14.039	.000
ConditionVisual	.866	.057	15.305	.000
Trial	-.018	.038	-.471	.637
DIF	.055	.137	.403	.687
DDF	-.034	.128	-.262	.794
EOT	-.006	.079	-.071	.943
ConditionCaption:Trial	.314	.057	5.523	.000
ConditionVisual:Trial	.334	.057	5.874	.000
ConditionCaption:DIF	-.730	.123	-5.916	.000
ConditionVisual:DIF	.052	.123	.423	.672
ConditionCaption:DDF	.422	.114	3.691	.000
ConditionVisual:DDF	-.018	.115	-.160	.873
ConditionCaption:EOT	.039	.071	.555	.579
ConditionVisual:EOT	-.257	.071	-3.624	.000
Trial:DIF	-.011	.083	-.133	.895
Trial:DDF	.012	.078	.152	.879
Trial:EOT	-.023	.047	-.489	.625
ConditionCaption:Trial:DIF	-.096	.123	-.781	.435
ConditionVisual:Trial:DIF	.005	.124	.038	.970
ConditionCaption:Trial:DDF	.074	.115	.639	.523
ConditionVisual:Trial:DDF	.003	.115	.022	.982
ConditionCaption:Trial:EOT	-.015	.071	-.211	.833
ConditionVisual:Trial:EOT	.006	.071	.084	.933
