# Supplementary material for: The Role of Alexithymia in Social Learning and Feedback-Driven Social Inferences
Source: Comput Psychiatr. 2026 Mar 19;10(1):35–57. doi: 10.5334/cpsy.153 (PMC13004067; doi:10.5334/cpsy.153)
Supplement: Supplementary Material File 4. — Detailed results from reinforcement learning model analyses. [file cpsy-10-1-153-s4.pdf]

Reinforcement Learning Models

The six models differed in two respects: (1) whether the learning rate (α) was shared across conditions or allowed to vary by condition, (2) whether learning was symmetric or split by the sign of the prediction error, (3) whether initial expected values (priors) were fixed at .5 or freely estimated as a parameter.

For the first sets of reinforcement learning (RL) models, we used a single learning rate to update the values associated with both the caption and the visual target (condition) for each participant. To further examine the potential differences in learning dynamics across cues, we implemented an alternative two-learning-rate model, in which separate learning rates were estimated for the visual and caption targets. In addition, for each of these sets, we also fit a variant with separate learning rates for positive and negative prediction errors (α^+ and α^-; prediction errors defined as $r_t - Q_{i, t-1}$). Finally, we fit an additional variant in which the initial values $Q_{i, 0}$ were freely estimated, allowing them to capture participants' priors. Thus, model-1 includes a single learning rate (α), and initial values for options are fixed to .5. Model-2 includes a single alpha for both conditions, but separate learning rates for positive and negative prediction errors (α^+, α^-). Model-3 includes a single alpha for both conditions, but the initial values of choices are freely estimated. Model-4 includes a separate learning rate per condition (α_{visual} and $\alpha_{caption}$) and initial values for options are fixed to .5. Model-5 contains four different learning rates, corresponding to different conditions and prediction errors (α^-_{visual}, α^+_{visual}, $\alpha^-_{caption}$, $\alpha^+_{caption}$), and initial values for options are fixed to .5. Model-6 contains a separate learning rate per condition (α_{visual} and $\alpha_{caption}$) and initial values for each option are also freely estimated. Results

regarding model-4 are presented in the main text. Thus, here, we will present the results regarding the other models.

Model 1: Same Learning Rate for targets, considering the initial expected values as 0.5

$Q_{i,0} = 0.5$

$\delta_t = (r_t - Q_{i,\, t-1})$

$Q_{i,t} = Q_{i,\, t-1} + \alpha_i \times \delta_t$

$P_{i,t} \approx exp(\beta(Q_{i,t})) \,/\, \Sigma_{i=1}^{k} exp\, (\beta(Q_{i,t}))$

Model 2: Same Learning Rate for targets, considering the initial expected values as 0.5, but different learning rates for positive and negative prediction errors

$Q_{i,0} = 0.5$

$\delta_t = (r_t - Q_{i,\, t-1})$

$Q_{i,t} = Q_{i,\, t-1} + \alpha_i^{+} \times \delta_t \,;\, if\, \delta_t > 0$

$Q_{i,t} = Q_{i,\, t-1} + \alpha_i^{-} \times \delta_t \,;\, if\, \delta_t < 0$

$P_{i,t} \approx exp(\beta(Q_{i,t})) \,/\, \Sigma_{i=1}^{k} exp\, (\beta(Q_{i,t}))$

Model 3: Same learning rate for targets, freely estimating initial expected values

$Q_{i,0}$: *freely estimated as a parameter subject to the constraint*

$\sum_{i=1}^{2} Q_{i,0} = 1$

$\delta_t = (r_t - Q_{i,\, t-1})$

$Q_{i,t} = Q_{i,\, t-1} + \alpha_i \times \delta_t$

$P_{i,t} \approx exp(\beta(Q_{i,t}))\ /\ \Sigma_i^k{}_{=1}\ exp\ (\beta(Q_{i,t}))$

Model 4: Different Learning Rate for targets, considering the initial expected values as 0.5

$Q_{i,0}^{k} = 0.5$

$\delta_t^k = (r_t^k - Q_{i,t-1}^k)$

$Q_{i,t}^k = Q_{i,t-1}^k + \alpha_i^k \times \delta_i^k$

$P_{i,t}^k \approx exp(\beta(Q_{i,t}^k))\ /\ \Sigma_i^k{}_{=1}\ exp\ (\beta(Q_{i,t}^k))$

Model 5: Different Learning Rate for targets, considering the initial expected values as 0.5, but different learning rates for positive and negative prediction errors

$Q_{i,0}^{k} = 0.5$

$\delta_t^k = (r_t^k - Q_{i,t-1}^k)$

$Q_{i,t}^{k} = Q_{i,t-1}^{k} + \alpha_{i}^{k+} \times \delta_{i}^{k}$; if $\delta_{i}^{k} > 0$

$Q_{i,t}^{k} = Q_{i,t-1}^{k} + \alpha_{i}^{k-} \times \delta_{i}^{k}$; if $\delta_{i}^{k} < 0$

$P_{i,t}^{k} \approx exp(\beta(Q_{i,t}^{k})) \,/\, \Sigma_{i=1}^{k} exp(\beta(Q_{i,t}^{k}))$

Model 6: Different Learning Rate for targets, freely estimating initial expected values

$Q_{i,0}^{k}$: *freely estimated as a parameter, subject to the constraint*

$\sum_{i=1}^{2} Q_{i,0}^{k} = 1$

$\delta_{t}^{k} = (r_{t}^{k} - Q_{i,t-1}^{k})$

$Q_{i,t}^{k} = Q_{i,t-1}^{k} + \alpha_{i}^{k} \times \delta_{i}^{k}$

$P_{i,t}^{k} \approx exp(\beta(Q_{i,t}^{k})) \,/\, \Sigma_{i=1}^{k} exp(\beta(Q_{i,t}^{k}))$

Where *i* denotes to response options, *t* denotes to trial number, and *k* denotes to conditions/targets.

Model Comparison

To assess model performance, we compared them using BIC. Model 4 with separate learning rates for targets showed significantly lower BIC compared to all other models, except model 6 (*p's* $< .05$). Among these two models, BIC was lower in model 4 (ΔBIC = .805). Thus, we chose model 4 to continue with in the main text. Yet, results regarding the other models are also presented in this supplementary. In general, irrespective of the model used, we found no significant difference between groups. Detailed BIC results are presented in Table S1.

Table S1 Mean and standard deviation of Bayesian Information Criterion (BIC) values for each model

Model	Mean
Model 1	111.930
Model 2	115.659
Model 3	115.372
Model 4	109.161
Model 5	116.666
Model 6	109.966

Range of parameters

The learning rate parameter (α) was constrained within the interval [0, 1]. To determine the optimal upper bound for the temperature parameter (β), we fitted the model using several ranges of β values (1, 5, 10, 15, 20, 25) and compared the resulting BIC values. The model with β ranging from 0 to 20 yielded the lowest BIC (M = 109.162, SD = 29.573) and was therefore selected for all subsequent analyses. Importantly, the key findings remained consistent across the different β ranges tested.

Model 1: Single Learning Rate for targets

Since model-1 includes a single learning rate, we conducted a t-test to examine the group differences and found no significant difference, $t(58) = .212$, $p = .833$.

Model 2: Single Learning Rate for Targets, Considering Positive and Negative learning Rates

A 2 × 2 mixed ANOVA was conducted on learning rates extracted from the Q-learning model 2, with group (HA, LA) as the between-subjects factor and Types of learning (α^{+}, α^{-}) as the within-subjects factor. The analysis revealed no significant interaction between group and type of learning. We found no significant interaction or main effect.

Table S2 Model-2: Mixed ANOVA Examining the Effects of Group and Prediction Error Type on Learning Rate.

Measure	*df*	*F*	*P*	*η2*
Prediction Error	1, 58	.014	.906	.001
Group	1, 58	.628	.431	.011
Prediction Error*Group	1, 58	1.149	.288	.019

Note. Prediction Error refers to the type of learning, where different alpha rates are used for learning from positive and negative prediction errors.

Model 3: Single Learning Rate for Targets, Considering Prior as a parameter

Since model-3 includes a single learning rate, we conducted a t-test to examine the group differences and we found no significant difference either in learning rate, $t(58) = .051$, $p = .251$, or prior, $t(58) = .513$, $p = .293$.

Model 5: Separate Learning Rates for Targets and Considering Positive and Negative learning Rates

A 2 × 2 × 2 mixed ANOVA was conducted on learning rates extracted from the Q-learning model 5, with group (HA, LA) as the between-subjects factor, and Condition (Visual, Caption) and Types of learning (α^+, α^-) as the within-subjects factors. We found no significant interaction or main effect.

Table S3 Model-5: Mixed ANOVA Examining the Effects of Group, Condition, and Prediction Error Type on Learning Rate

Measure	df	F	P	η2
Condition	1, 58	.064	.801	.001
Prediction Error	1, 58	.186	.668	.003
Group	1, 58	.161	.690	.003
Condition * Prediction Error	1, 58	.186	.668	.003
Condition * Group	1, 58	.042	. 839	.003
Prediction Error * Group	1, 58	1.585	.216	.026
Condition * Prediction Error*Group	1, 58	1.585	.216	.026

Model 6: Separate Learning Rates for Targets and Considering Positive and Negative learning Rates

A 2 × 2 mixed ANOVA was conducted on learning rates extracted from the Q-learning model 6, with group (HA, LA) as the between-subjects factor, and Condition (Visual, Caption) as the within-subjects factor. We found no significant interaction or main effect of Group, but a significant effect of Condition, where learning rate for the caption-correct target was higher.

Table S4 Model-6: Mixed ANOVA Examining the Effects of Group and Condition on Learning Rate.

Measure	*df*	*F*	*P*	*η2*
Condition	1, 58	4.939	.030	.078
Group	1, 58	2.803	.099	.046
Prediction Error*Group	1, 58	3.356	.072	.055

Parameter Recovery

We performed a parameter recovery analysis on our best-fitting model to assess the identifiability of its parameters. Specifically, we used various combinations of the model's key parameters (visual learning rate, caption learning rate, and inverse temperature) to generate synthetic datasets. Ten values for each learning rate (ranging from .1 to .9) and twenty values for the inverse temperature (ranging from 1 to 19) were used (2000 sets total).

To simulate participants' responses, we added noise to each parameter (a standard normal distribution scaled by .1 for learning rates and by 1 for inverse temperature; see Cutler et al., 2021; Lockwood et al., 2019). Parameter values were constrained within the predefined bounds of the model (learning rates: [0, 1]; inverse temperature: [0, 20]). The small offset from the boundaries was applied to prevent redundant parameter values when adding noise (for example, if the learning rate equals 1 and positive noise is added, it would be clipped to 1 repeatedly).

For each parameter combination, we generated ten experimental simulations which were differing in trial order. Furthermore, parameter re-estimation was performed 50 times using different initial values to avoid local optima.

The results approved our model's ability to recover all the parameters in our best-fitting model by showing strong correlations between the real and recovered parameters over the

different combinations and a wide range of values (Figure S1). Moreover, correlations were higher or comparable to previous studies in the field (Barnby et al., 2022; Cutler et al., 2021; Steinke et al., 2020; Toyama et al., 2023).

Figure S1 Correlation Matrix of the Relationship Between Real and Recovered Parameters.

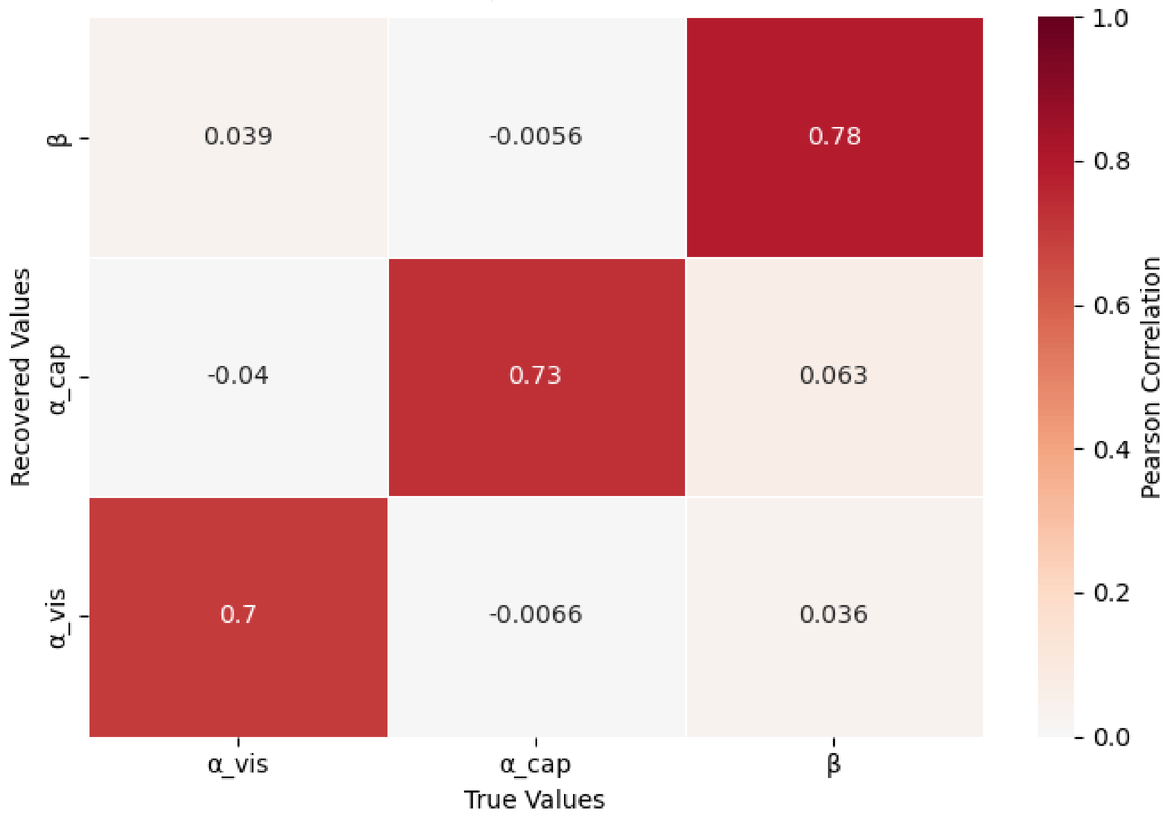

References

Cutler, J., Wittmann, M. K., Abdurahman, A., Hargitai, L. D., Drew, D., Husain, M., & Lockwood, P. L. (2021). Ageing is associated with disrupted reinforcement

learning whilst learning to help others is preserved. *Nature communications*, *12*(1), 4440. https://doi.org/10.1038/s41467-021-24576-w

Lockwood, P. L., Klein-Flügge, M., Abdurahman, A., & Crockett, M. J. (2019). Neural signatures of model-free learning when avoiding harm to self and other. *BioRxiv*, 718106. https://doi.org/10.1101/718106

Barnby, J. M., Mehta, M. A., & Moutoussis, M. (2022). The computational relationship between reinforcement learning, social inference, and paranoia. *PLoS Computational Biology*, *18*(7), e1010326. https://doi.org/10.1371/journal.pcbi.1010326

Steinke, A., Lange, F., & Kopp, B. (2020). Parallel model-based and model-free reinforcement learning for card sorting performance. *Scientific reports*, *10*(1), 15464. https://doi.org/10.1038/s41598-020-72407-7

Toyama, A., Katahira, K., & Kunisato, Y. (2023). Examinations of biases by model misspecification and parameter reliability of reinforcement learning models. *Computational Brain & Behavior*, *6*(4), 651-670. https://doi.org/10.1007/s42113-023-00175-4
